# Rootless tephra stratigraphy and emplacement processes

**DOI:** 10.1007/s00445-016-1086-4

**Published:** 2017-01-10

**Authors:** Christopher W. Hamilton, Erin P. Fitch, Sarah A. Fagents, Thorvaldur Thordarson

**Affiliations:** 1 Lunar and Planetary Laboratory, University of Arizona, 1629 E. University Blvd., Tucson, AZ 85721 USA; 2 Department of Geology and Geophysics, University of Hawaii, Honolulu, HI USA; 3 Hawaii Institute of Geophysics and Planetology, University of Hawaii, Honolulu, HI USA; 4 Faculty and Institute of Earth Sciences, University of Iceland, Reykjavík, Iceland

**Keywords:** Volcanic rootless cones, Pseudocraters, Lava, Water, Phreatomagmatic, Iceland

## Abstract

Volcanic rootless cones are the products of thermohydraulic explosions involving rapid heat transfer from active lava (fuel) to external sources of water (coolant). Rootless eruptions are attributed to molten fuel–coolant interactions (MFCIs), but previous studies have not performed systematic investigations of rootless tephrostratigraphy and grain-size distributions to establish a baseline for evaluating relationships between environmental factors, MFCI efficiency, fragmentation, and patterns of tephra dispersal. This study examines a 13.55-m-thick vertical section through an archetypal rootless tephra sequence, which includes a rhythmic succession of 28 bed pairs. Each bed pair is interpreted to be the result of a discrete explosion cycle, with fine-grained basal material emplaced dominantly as tephra fall during an energetic opening phase, followed by the deposition of coarser-grained material mainly as ballistic ejecta during a weaker coda phase. Nine additional layers are interleaved throughout the stratigraphy and are interpreted to be dilute pyroclastic density current (PDC) deposits. Overall, the stratigraphy divides into four units: unit 1 contains the largest number of sediment-rich PDC deposits, units 2 and 3 are dominated by a rhythmic succession of bed pairs, and unit 4 includes welded layers. This pattern is consistent with a general decrease in MFCI efficiency due to the depletion of locally available coolant (i.e., groundwater or wet sediments). Changing conduit/vent geometries, mixing conditions, coolant and melt temperatures, and/or coolant impurities may also have affected MFCI efficiency, but the rhythmic nature of the bed pairs implies a periodic explosion process, which can be explained by temporary increases in the water-to-lava mass ratio during cycles of groundwater recharge.

**Electronic supplementary material:**

The online version of this article (doi:10.1007/s00445-016-1086-4) contains supplementary material, which is available to authorized users.

## Introduction

Volcanic rootless cones (VRCs) are the products of explosive lava–water interactions and are commonly associated with the flow of lava into marshes, lacustrine basins, and littoral environments, as well as over glacial outwash plains, snow, and ice (Thorarinsson 1951, 1953; Moore and Ault 1965; Fisher 1968; Jurado-Chichay et al. 1996; Thordarson et al. 1998; Fagents et al. 2002; Fagents and Thordarson 2007; Reynolds et al. 2015). Terrestrial rootless cone groups cover areas of up to ~150 km^2^ and generally include numerous cratered cones ranging from 1 to 35 m in height and 2 to 450 m in diameter (Fagents and Thordarson 2007). Rootless cone morphologies and patterns of spatial organization are hypothesized to strongly depend upon lava emplacement processes and a balance between the availability and utilization of lava (fuel) and groundwater (coolant), which influences the efficiency of molten fuel–coolant interactions (MFCIs; Colgate and Sigurgeirsson 1973; Drumheller 1979; Sheridan and Wohletz 1981, 1983; Wohletz and Sheridan 1981, 1983; Wohletz 1983, 1986, 2002; Zimanowski et al. 1991, Zimanowski 1998).

The general sequence of formation for rootless cone groups in Iceland involves the initial emplacement of lava over deformable water-bearing sediments (e.g., glacial outwash plains, marshes, or lacustrine sediments), followed by the gradual thickening of the flow through the process of inflation (Hon et al. 1994; Hamilton et al. 2013). Initially, the lava–substrate interface is insulated by the development of a stable basal crust, but as the flow thickens and increasingly exerts pressure on the underlying sediments, the lava may subside into the substrate and develop cracks in the basal crust that allow lava within the molten core to come into direct contact with the waterlogged sediments below (Fagents and Thordarson 2007; Hamilton et al. 2010a, 2010b). This process can initiate cycles of dynamic mixing and explosive lava–water interactions that eject substrate sediments and overlying lava (including both molten material and solidified crust) and construct a rootless cone from the fragmental debris. At any given rootless eruption site, these explosion cycles may continue until the water and/or lava supply diminishes below a critical threshold, at which point cone construction ceases. The resulting rootless tephra sequence (RTS) typically includes a vent-proximal cone facies (dominated by ballistic ejecta), which grades into a distal sheet facies (dominated by tephra fall); however, in the vent-proximal to medial region, both of these emplacement processes combine to form a platform facies (Hamilton et al. 2010a). The geometry of the VRC will tend to be radially symmetric about its associated rootless explosion site, but the final morphology of a volcanic rootless cone group may be complicated by the deposition of tephra from other explosions that may initiate concurrently or sequentially within other parts of the active lava flow field.

The purpose of this work is to combine detailed stratigraphic observations with quantitative descriptions of tephra grain-size distributions to establish an improved baseline for studying rootless cone architecture, deposit facies, and eruption mechanisms. These investigations are important because of their implications for better understanding the geologic hazards associated with explosive magma–water interactions, and for determining the paleo-environmental significance of VRCs on Earth and Mars (Frey et al. 1979; Frey and Jarosewich 1982; Greeley and Fagents 2001; Lanagan et al. 2001; Head and Wilson 2002; Fagents et al. 2002; Fagents and Thordarson 2007; Jaeger et al. 2007; Hamilton et al. 2010b, 2010c, 2011).

### Molten fuel–coolant interaction theory

Phreatomagmatic eruptions involve the intimate mingling of silicate melt and external water (Morrisey et al. 2000) and therefore differ from phreatic (“steam”) explosions caused by heating water through a stable interface. Phreatomagmatic and phreatic eruptions contribute directly to the formation of VRCs (including littoral cones), tuff cones, tuff rings, maars, some stratovolcano deposits, submarine eruptions, and ice-contact volcanoes (Wohletz 1986; Kokelaar 1986; Guðmundsson et al. 1997; Hickson 2000; Morrisey et al. 2000; Lescinsky and Fink 2000; Greeley and Fagents 2001; Fagents et al. 2002; Andrews 2003; Belousov et al. 2011; Schipper et al. 2013). The thermodynamics of phreatomagmatic eruptions are described by the theory of MFCIs, which includes four principal stages: (1) hydrodynamic premixing, (2) triggering, (3) fine fragmentation, and (4) vaporization and expansion (Wohletz 1983, 1986, 2002; Zimanowski et al. 1991; Zimanowski 1998; Morrisey et al. 2000; Büttner et al. 2002). Explosive MFCI eruptions may involve numerous cycles of premixing, triggering, fine fragmentation, and expansion, whereas non-explosive MFCIs tend to terminate at the hydrodynamic premixing or triggering phase (Morrisey et al. 2000).

Grain-size distributions and tephra dispersal patterns largely depend upon phreatomagmatic interaction efficiency, such that with increasing efficiency, more thermal energy converts to mechanical energy, which increases fragmentation and dispersal range (Morrisey et al. 2000). The efficiency of a phreatomagmatic interaction is termed the conversion ratio (CR), which is defined as the work of the system, *W*_sys_, divided by the thermal energy of the magma (Wohletz 1986):

1 $$ CR=\frac{W_{sys}}{m_{\mathrm{m}}{C}_{\mathrm{m}}\left({T}_{\mathrm{m}}-{T}_{ref}\right)}=\frac{\Delta KE+\Delta PE+{p}_0\Delta {V}_{sys}}{m_{\mathrm{m}}{C}_{\mathrm{m}}\left({T}_{\mathrm{m}}-{T}_{ref}\right)} $$

where *m*_m_ is the mass of the magma, *C*_m_ is the specific heat of the melt, *T*_m_ is the absolute temperature of the melt, *T*_ref_ is the reference temperature (298 K), ΔKE is the change in kinetic energy, ΔPE is the change in potential energy, *p*_0_ is the initial pressure, and Δ*V*_sys_ is the change in the volume of the system. To achieve the most efficient reactions, complete thermal equilibrium is necessary and requires uniform melt fragments with a diameter of approximately 1 μm (Wohletz 1986). However, in nature, poorly sorted phreatomagmatic deposits with juvenile grain sizes ranging from ash to lapilli indicate that the efficiency of phreatomagmatic interaction is well below the theoretical limit, with natural phreatomagmatic explosions generally having an efficiency of 10% or less (Wohletz 1986).

Laboratory experiments provide valuable insight into MFCI theory and its application to phreatomagmatic eruptions, but the restricted scale of these experiments limits the spectrum of volcanic processes that can be represented. Rootless eruptions provide natural analogs for investigating larger MFCIs because explosive interactions involving outgassed lava and groundwater are unaffected by the volumetric expansion of juvenile magmatic volatiles, as is typically the case in primary volcanic conduits. In this study, we focus on the bedding characteristics and grain-size distributions within rootless cone deposits to develop a better understanding of mixing conditions, phreatomagmatic interaction efficiency, and emplacement mechanisms of tephra generated during natural MFCI explosions.

### Geological context of the study area

**Fig. 1:**
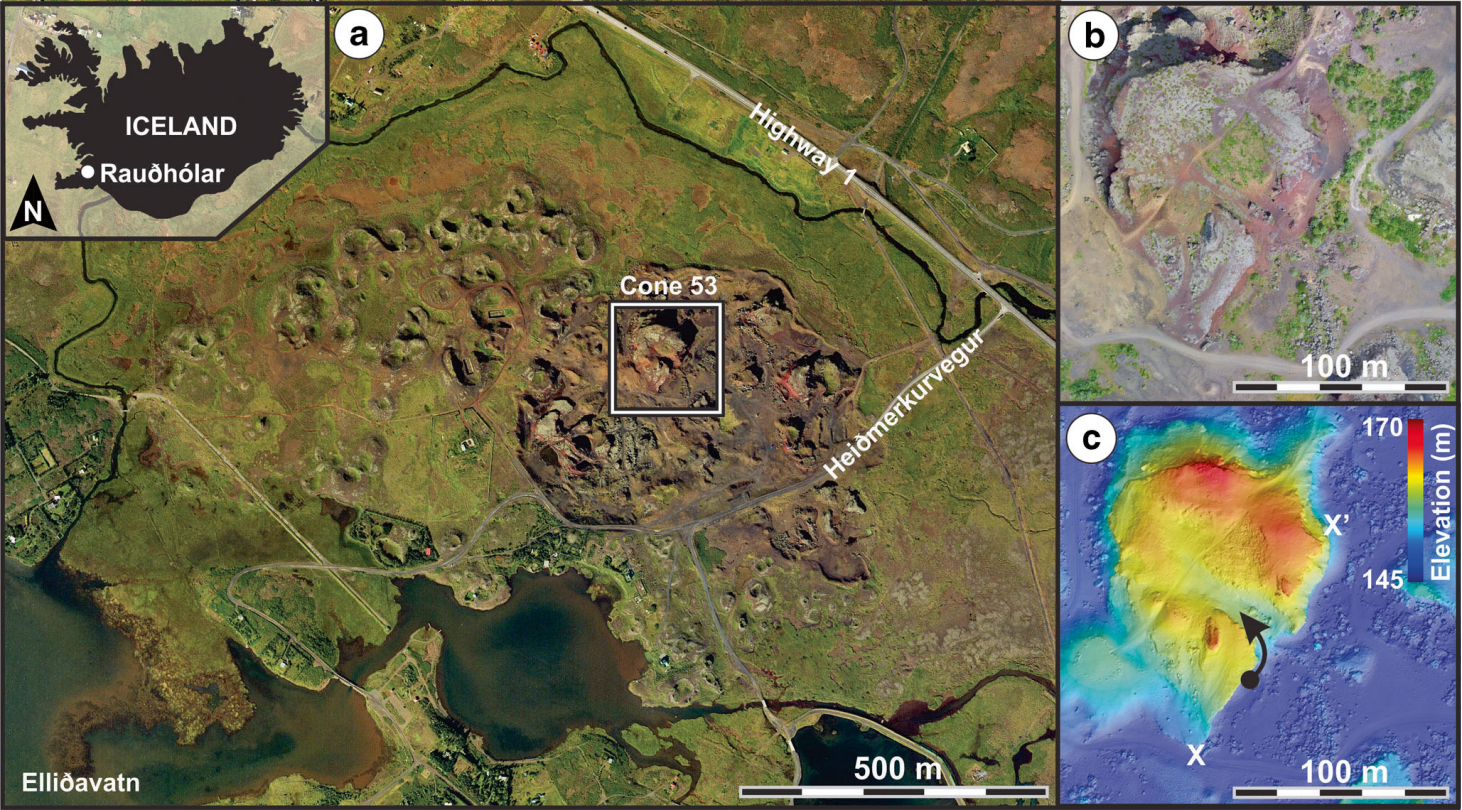
**a** Rauðhólar located within the Elliðaá lava flow, north of the present-day Lake Elliðavatn. The *inset* shows the location of the study site within southwestern Iceland. This study focuses on the stratigraphy within Cone 53. **b** and **c**, respectively, show an orthoimage mosaic and digital terrain model of Cone 53 that were derived from unmanned aerial vehicle (UAV) imagery using multi-view stereo-photogrammetry (Hamilton et al. 2015). Sampling of Rootless Tephra Sequence 2 (RTS2) was conducted along a composite transect marked with the *filled black circle* (64.094893° N, −21.751814° E) and extending to the end of the arrow (64.095103° N, −21.752074° E) shown in **c**. The cross section *X–X’* is shown in Fig. 2b. North is up in panels **a**, **b**, and **c**

The Rauðhólar rootless cone group (hereafter referred to as Rauðhólar) is located within the ~5200-year-old (Sinton et al. 2005) Elliðaá lava flow (Elliðaáhraun in Icelandic) in the Western Volcanic Zone of Iceland (Fig. 1a). The Elliðaá lava flow is interpreted to be an early phase of the Leitin lava shield, which is comprised of pāhoehoe with an olivine tholeiite composition (Rossi and Gudmundsson 1996; Rossi 1997; Sinton et al. 2005). Pāhoehoe flow fields grow by lava inflation (Walker 1991; Hon et al. 1994; Hamilton et al. 2013), which implies that Elliðaá lava was transported away from the source region through a network of thermally insulated internal pathways (i.e., lava tubes).

Distal tephra deposits from these rootless eruption sites combine to form a platform facies that covers an area of ~1.2 km^2^ and rises ~5 m above the surface of the Elliðaá lava flow. Rootless cone morphologies within Rauðhólar range from meter-scale hornito-like forms located near the margins of the group to larger cratered tephra cones located near the center of the group. The largest of these VRCs is 212 m in basal diameter and rises ~22 m above the surrounding lava surface. The H-37 drill hole penetrates through the Elliðaá lava flow field, just north of Rauðhólar, and provides information on the local stratigraphic succession, which includes a ~7-m-thick layer of the Elliðaá lava directly above a >1-m-thick layer of lacustrine sediment and diatomaceous siltstone, which thickens toward the south and is inferred to underlie the paleo-lake basin now occupied by Rauðhólar (Tómasson et al. 1977).

**Fig. 2:**
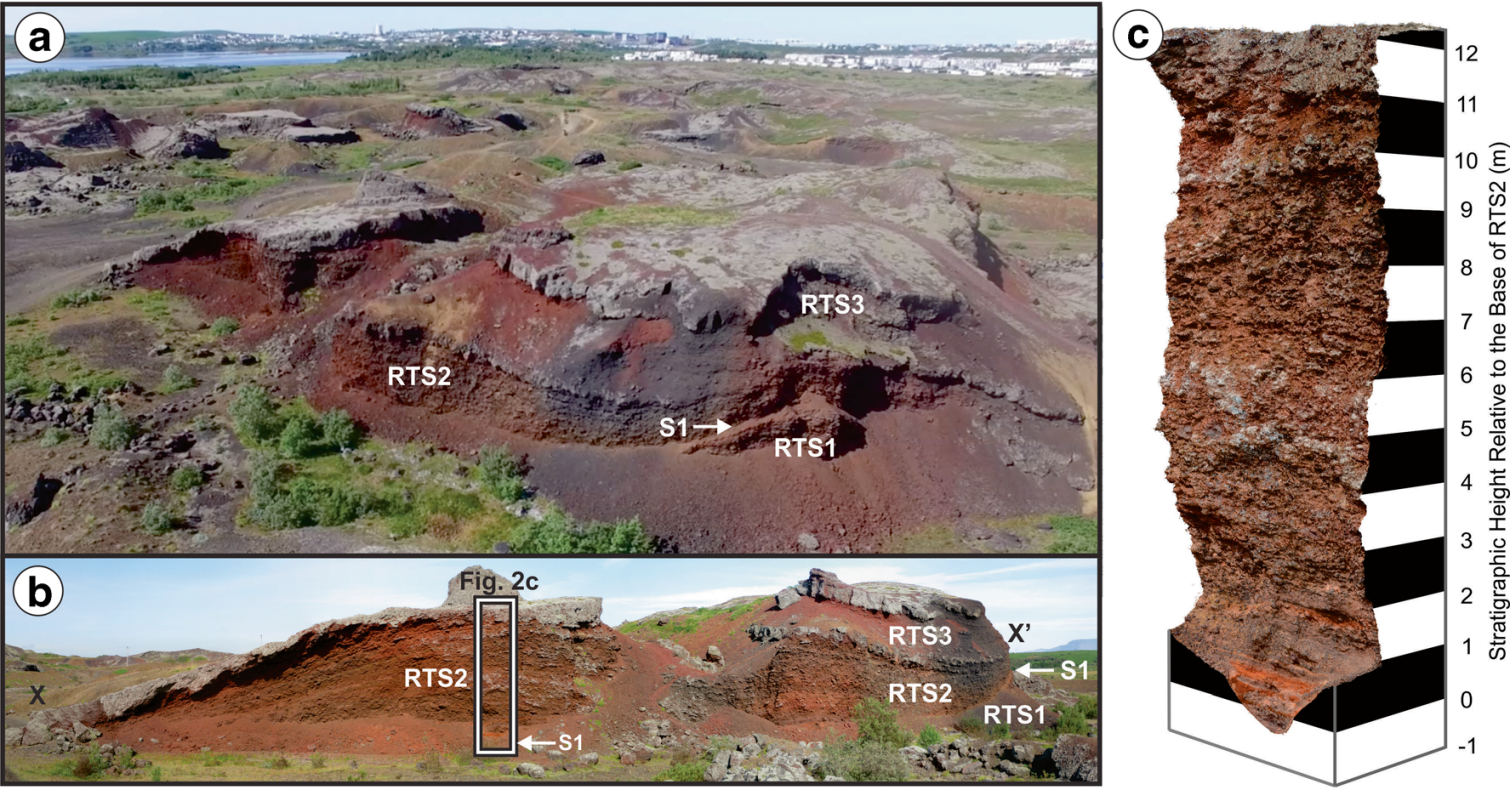
**a** Oblique aerial view and **b** ground-based photographic mosaic of Cone 53 showing the locations of Rootless Tephra Sequence (RTS) 1, 2, and 3, as well as the marker horizon S1. **c** The primary focus of this study is on the southern cross section though RTS2, which opens with the ochre-colored mud-rich S1 layer (at a stratigraphic height of 0 m) and terminates in a gray welded spatter layer at the top of the sequence

Rauðhólar was extensively quarried during the early- to mid-twentieth century, which destroyed the original morphology of the rootless cone group, but generated excellent windows into the stratigraphy of the VRCs. Fortunately, von Komorowicz (1912) recorded the initial arrangement of cones and craters, and this study focuses on an exceptional exposure through one of the large VRCs located near the center of Rauðhólar (Fig. 1), which von Komorowicz (1912) labeled as “Cone 53” in his map. The NNW–SSE-trending cross section into Cone 53 extends ~150 m and includes a southern and a northern component, divided by a partially quarried section in the middle (Fig. 2). The precise location of the rootless vent(s) that formed Cone 53 cannot be determined due to quarrying operations, but according to the map by von Komorowicz (1912), the centroid of the closest exposed crater was ~20 m away from the location of our stratigraphic section.

The archetypical RTS examined within this study (i.e., Rootless Tephra Sequence 2, RTS2) exhibits an approximate mirror symmetry with respect to the inferred vent location, and the northern part of the deposit drapes the flank of an older cone, which we term Rootless Tephra Sequence 1 (RTS1). Relative to RTS1, layers within RTS2 have a discordant bedding geometry and contrasting thinning direction, which implies RTS1 and RTS were erupted from different sources. The basal layer within RTS2, labeled S1 in Fig. 2, contains abundant ochre-orange lacustrine sediment (hereafter also referred to as “mud”), and it serves as a marker bed for identifying the beginning sequence. The top of RTS2 is composed of a welded spatter layer. Material above this welded spatter layer (i.e., Rootless Tephra Sequence 3, RTS3) has been largely removed by quarrying operations in the southern cross section but remains intact in the northern part of the exposure. Cone 53 is therefore a composite structure composed of at least three onlapping tephra sequences from distinct, but nearby, rootless eruption sites. This study focuses on the middlemost of these tephra sequences, RTS2, which is fully exposed in the southern cross section (Fig. 2).

## Methods

Following an initial investigation by Morrissey and Thordarson (1991), fieldwork for this project developed over a period from 2002 to 2016. During that time, the 13.55-m-thick composite stratigraphic section through RTS2 was logged at the centimeter scale and divided into 68 layers. Sixty-four layers were sampled for grain size, but welding prohibited the collection of a representative grain-size sample from the remaining four layers.

For each grain-size sample, the largest clast did not exceed 5% of the total sample mass, and material was processed using a combination of field-based and laboratory methods to generate composite statistics. In the field, material was sieved at intervals of 1*ϕ*, from 4 mm (−2*ϕ*) to 512 mm (−9*ϕ*), and weighed using an electronic balance with a precision of 0.1 g. Clasts with an intermediate axis <4 mm (*ϕ* > −2) were returned to the laboratory, dried at 110 °C for 24 h, and reweighed, which enabled the weight of the field samples to be corrected to a dry mass by assuming that both samples initially contained the same proportion of water. Additionally, the dried laboratory samples were sieved by hand at intervals of 0.5*ϕ*, from 4 mm (−2*ϕ*) to 32 μm (5*ϕ*), and weighed using an electronic balance with a precision of 0.01 g. Final grain-size statistics were calculated using the Folk and Ward method in the GRADISTAT program (Version 8), in accordance with the procedures recommended by Blott and Pye (2001).

Most samples with an intermediate axis <1 mm (i.e., *ϕ* > 0) included indurated aggregates of diatomaceous lacustrine sediment mixed with a minor component of basaltic tephra. We term these aggregates “mud clots,” and for the grain-size analyses, the mud clots were not mechanically crushed or altered. However, to account for the abundance of lacustrine sediment (i.e., mud) in each sample, the relative proportions of the mud clots versus basaltic tephra were visually estimated between −3.0*ϕ* and 4.5*ϕ* using a standard comparison chart for visual percentage estimation (Terry and Chilingar 1955).

Results of our grain-size analysis are reported using the following divisions (White and Houghton 2006): extremely fine ash (*ϕ* > 4.0), very fine to very coarse ash (4.0 ≥ *ϕ* > −1.0), fine to medium lapilli (−1.0 ≥ *ϕ* > −4.0), coarse lapilli (−4.0 ≥ *ϕ* > −6.0), and bombs (*ϕ* ≤ −6.0). All layers in the stratigraphy range from poorly sorted to very poorly sorted, which are defined as 1.0 ≤ *σ*_*ϕ*_ < 2.0 and *σ*_*ϕ*_ ≥ 2.0, respectively. Mean grain size and mean sorting are distinguished using an overbar symbol.

## Results

### Vertical stratigraphy and grain size

**Fig. 3:**
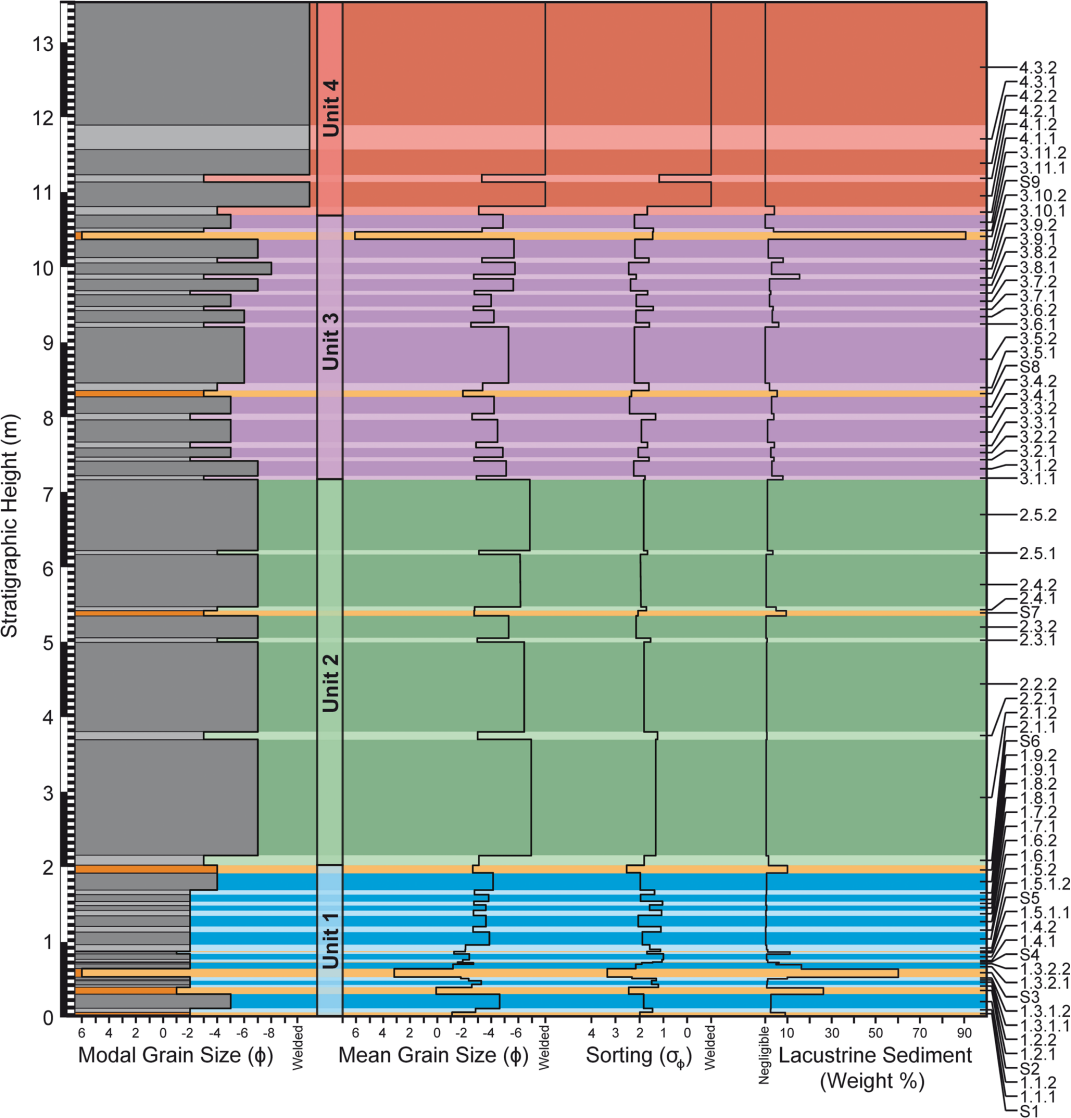
Stratigraphic log of RTS2 showing the modal grain size, mean grain size, sorting, and lacustrine sediment abundance within each of the 68 layers identified in the stratigraphy. These layers divide into four units, which are comprised of 28 bed pairs, nine S layers, and three mixed layers. In the *left-hand column*, lower bed pairs are shown in *light gray*; upper bed pairs are shown in *dark gray*, and S layers are shown in *orange*. For convenience throughout this study, units 1, 2, 3, and 4 are represented in *blue*, *green*, *violet*, and *red*, respectively. Unit names are also given using an *A*.*B*.*C*.*D*. notation, where *A* represents the unit number, *B* the bed-pair number within a unit, *C* whether or not a unit is a lower (i.e., 1) or upper (i.e., 2) bed pair, and where mixing occurs, *D* is used to further subdivide a bed pair into a lower (i.e., 1) or upper (i.e., 2) part, with one part being mixed and the other unmixed, depending on the local contact geometry. S layers are labeled *S* *1*–*S* *9*

The composite stratigraphic section through RTS2 has a total height of 13.55 m and passes through the thickest part of the deposit. The stratigraphy includes 68 layers, which we divide into four units (Fig. 3 and Appendix 1). The lowermost unit (i.e., unit 1) spans in stratigraphic height from 0 to 201 cm, unit 2 extends 201–721 cm, unit 3 extends 721–1081 cm, and unit 4 extends 1081–1355 cm.

The stratigraphy is dominated by a rhythmic succession of 28 bed pairs. Unit 1 includes nine bed pairs, unit 2 includes five bed pairs, unit 3 includes 11 bed pairs, and unit 4 includes 3 bed pairs—for a total of 56 individual layers. Each bed pair is composed of a fine-grained lower component and a coarser-grained upper component, which is assigned a name according to an *A*.*B*.*C*. notation, where *A* indicates the unit number (ranging from 1 to 4), *B* indicates the layer number within the unit (ranging up to 11), and *C* indicates whether or not the unit is the lower (i.e., 1) or upper (i.e., 2) member of each bed pair. There are nine additional mud-rich layers (labeled S1 through S9) that are interleaved and, in some cases, mixed with the bed pair deposits. Where bed-pair deposits are mixed with an S layer—this occurs only in unit 1—units are further subdivided using an *A*.*B*.*C*.*D*. notation, where *D* is used to distinguish between mixed and unmixed layers.

**Fig. 4:**
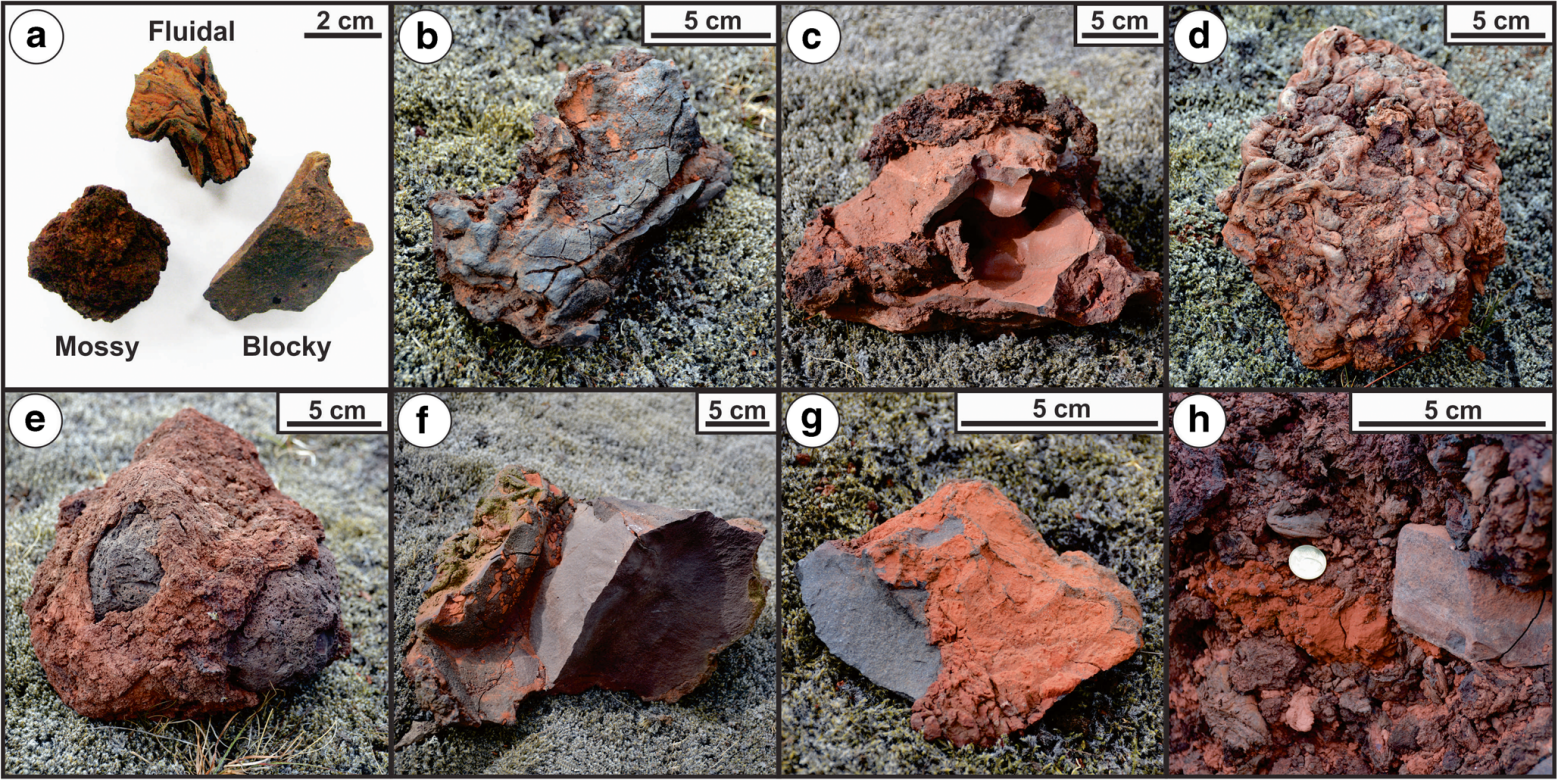
**a** Examples of fluidal, mossy, and blocky lapilli clasts. **b** Curvilinear fractures on the underside of a fluidal clast. **c** Hybrid fluidal–mossy clast with *red coloration* caused by a surface coating of fine lacustrine sediment. **d** Subrounded clast composed of an amalgamation of smaller fluidal and mossy clasts and lacustrine sediment. **e** Armored bomb including a rounded lithic interior and a coating of mossy lava material. **f** Another example of a armored bomb with an angular lithic block partially coated in a rind of fluidal lava with entrained clots of lacustrine sediment. **g** Lava-lithic fragment with a layer of “baked” lacustrine sediment (i.e., “mud”) fused to the surface. **h** A typical example of a mud clot to the left of a lithic bomb within RTS2 (n.b., a 22.5-mm-wide 100 Icelandic krónur coin is included for scale)

A rust-red color is typical for most VRC deposits within Rauðhólar (meaning “red hills” in Icelandic). RTS2 is also rust-red in color, except for the topmost unit (unit 4), which includes gray welded spatter layers, and the S layers, which tend to be ochre-orange (Figs. 1d and 2). Oxidation of the lava may play a minor role in generating this color, but the clasts are red primarily because they are coated with a thin film of red- to ochre-colored mud that is agglutinated onto their surfaces. Fitch et al. (2017) provided a detailed examination of the componentry of the layers within the RTS2 layer, but results are discussed here for the lapilli-size fraction to aid in the overall characterization of the stratigraphy. The majority (61–94%) of the clasts within the bed-pair layers exhibit fluidal morphologies (Fig. 4a). Fluidal morphologies are distinguished from mossy and blocky clasts (Fig. 4a) on the basis of shape and surface texture for clasts within the −4*ϕ* to −5*ϕ* (16–32 mm) size range. Fluidal clasts have ribbon-like to spindle-shaped forms with elongate structures resembling those of pulled taffy. Their surfaces are generally smooth, though in some cases can be rough, where coated by coarser grains of agglutinated sediment. In contrast, lapilli-sized mossy clasts range in abundance from 5 to 32% and tend to be less elongate and exhibit a rougher texture resembling the spinose surface of ‘a‘ā clinker. Lastly, lapilli-sized blocky clasts range in abundance from 1 to 25%, are more equant in shape, and have planar fracture faces that are typically covered in a fine layer of lacustrine sediment. The proportions of these three clast types do not vary significantly between the lower and upper bed-pair members within the same unit (Fitch et al. 2017), nor are there significant variations in the abundance of fluidal versus mossy material between units. However, there is a distinct decrease in the abundance of blocky clasts with increasing stratigraphic height. On average, juvenile clasts in the S layers have 63% fluidal, 27% mossy, and 9% blocky morphology (Fitch et al. 2017) and are the most similar to the bed pairs in unit 1. Additionally, juvenile clasts within RTS2 commonly exhibit curvilinear fracture networks that occur preferentially on the lower surface of the clasts (Fig. 4b). These fractures affect up to a quarter of all samples but are more common on fluidal clasts than mossy clasts, and they do not appear on the surfaces of the blocky clasts.

Both units 1 and 3 have considerably higher abundances of diatomaceous lacustrine sediment (6 and 7% mean mud content, respectively) than unit 2 (2% mean mud content). The form of the sediment ranges from unconsolidated grains and aggregates to weakly consolidated mud clots and hardened masses of lacustrine sediment. Larger clasts commonly exhibit complex mixing relationships with diatomaceous sediments and layers of fluidal or mossy material (Fig. 4c–f). These armored bombs typically include a nucleus of fluidal lava mixed with baked sediment, or an angular block of lithic lava that may have glassy selvage and/or baked lacustrine sediments adhered to the surface. Isolated mud clots are also common within RTS2 and typically range up to several centimeters in diameter (Fig. 4h).

Within units 1, 2, and 3, the mean grain sizes for the lower bed-pair members are −2.5*ϕ*, −3.0*ϕ*, and −2.9*ϕ*, respectively. These layers typically exhibit conformal (i.e., mantling) bedding structures and are all poorly sorted, with the mean sorting among lower bed-pair layers in unit 1 being slightly more developed (${\overline{\sigma}}_{\phi }$ = 1.3) than the sorting in units 2 and 3 (${\overline{\sigma}}_{\phi }$ = 1.6 for both units). The upper bed-pair layers in units 1, 2, and 3 have mean grain sizes of −3.5*ϕ*, −6.3*ϕ*, and −4.9*ϕ*, respectively. Upper bed-pair layers in units 1 and 2 are generally poorly sorted (${\overline{\sigma}}_{\phi }$ = 1.8 for both units), whereas upper bed-pair layers in unit 3 are very poorly sorted (${\overline{\sigma}}_{\phi }$ = 2.2). Units 1.3.1.2, 1.3.2.1, and 1.5.1.1 are excluded from the statistical calculations due to mixing with adjacent S layers, and statistical values are not reported for unit 4 due to the effects of welding. See Appendix 1 for a complete record of all of the grain-size information.

Interleaved with the bed pairs are nine mud-rich layers, designated S layers, which are ochre-orange in color (Figs. 2 and 3). These deposits include abundant quantities of diatomaceous lacustrine sediment, with a mean mud content of 24%, though it ranges up to 91% for S9. Some of these deposits (e.g., S3) can exhibit well-defined fine-scale internal laminations and low-angle cross-bedding as well as “pinch-and-swell” thickness variations that develop where the deposits are thin above local topographic obstacles and thicker above topographic depressions. These local thickness variations contrast with the conformal geometry of the bed-pair units described above. Thicker S layers, particularly in unit 1, also tend to exhibit asymmetrical depositional characteristics on the stoss and lee sides of high-standing topographic obstacles, such as bombs that protrude from underlying layers. Additionally, where coarse lapilli and bombs are present, the fine-scale internal structures of the S layers are typically deformed around these larger clasts. The mean grain size of the S layers is −0.3*ϕ* (coarse ash), though the deposits are very poorly sorted (${\overline{\sigma}}_{\phi }$ = 2.1). This poor degree of sorting reflects the bimodal nature of these layers, which includes a fine-grained mud-rich matrix that supports larger clasts (typically lapilli and rare bombs) and coarse-ash lenses. Except where otherwise noted, S layers have sharp contacts with adjacent units above and below.

#### Unit 1

**Fig. 5:**
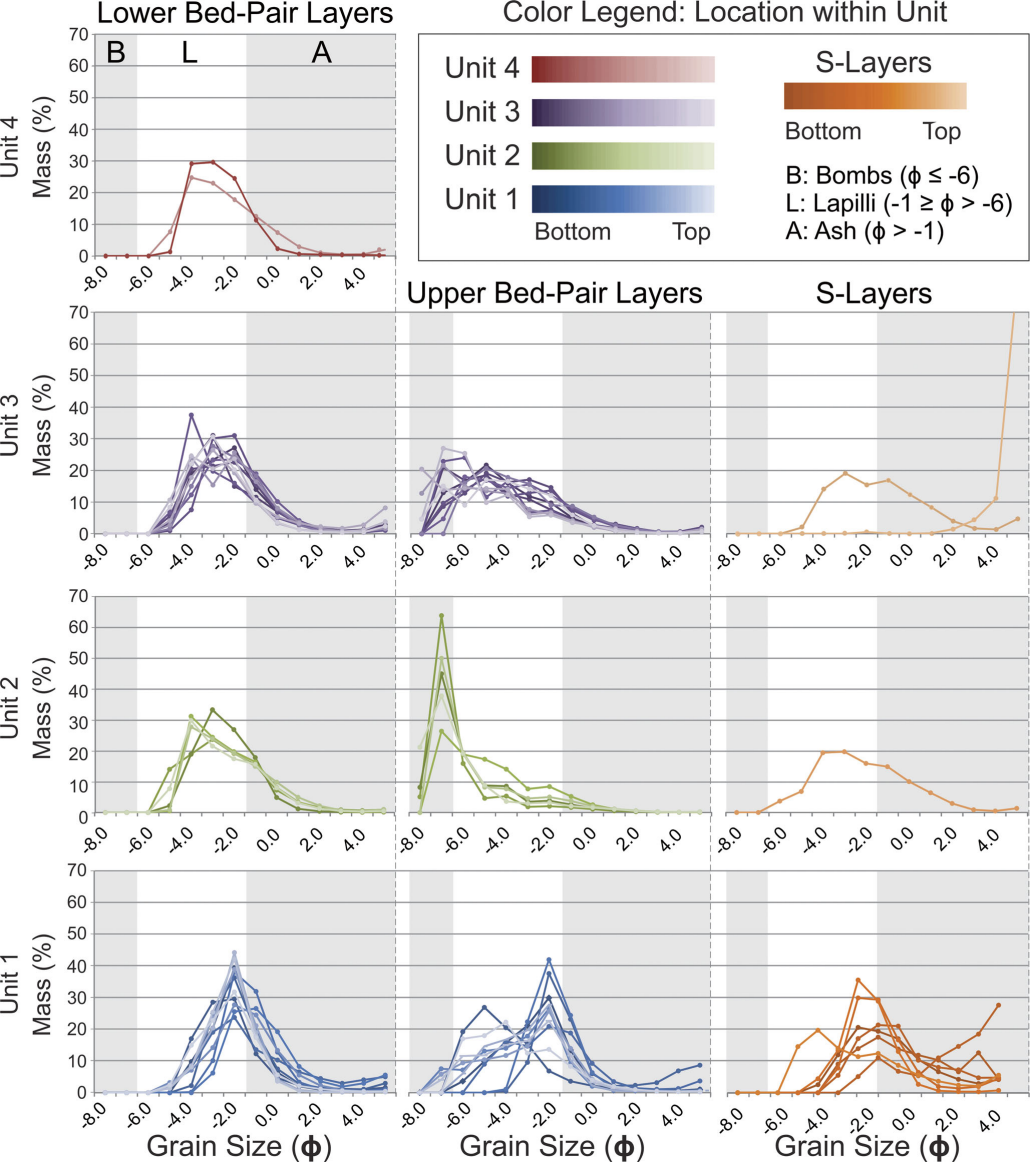
Composite grain-size plots for bed-pair layers within units 1–4 and the S layers. To facilitate the presentation, histograms have been converted over to stacked line plots. Unit 4 grain-size distributions are only shown for layers 4.1.1 and 4.2.1, due to the high degree of welding within the other layers in this unit

**Fig. 6:**
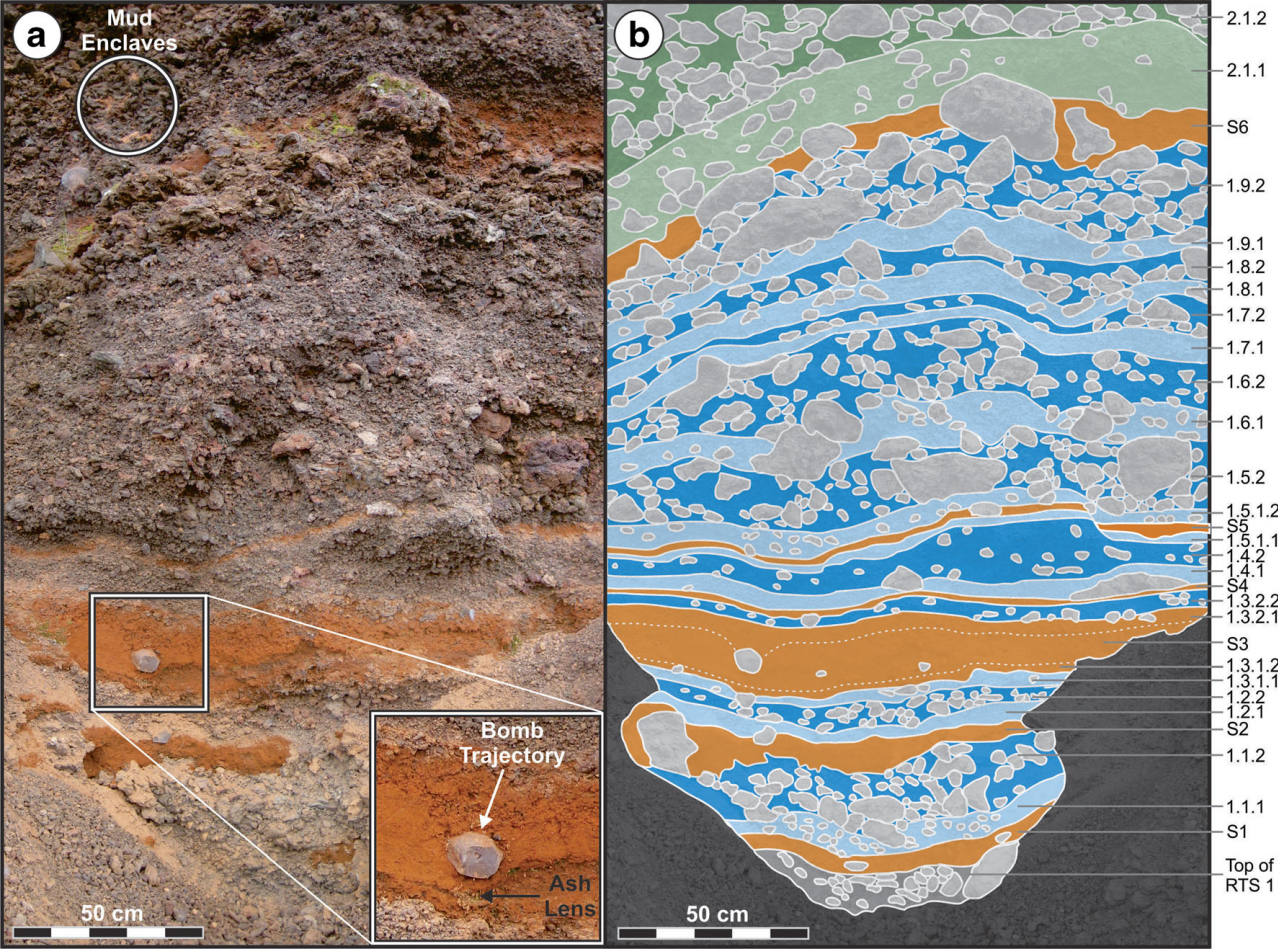
**a** Photographic mosaic of unit 1 showing details of the layering structure of the bed pairs and S layers. *Inset* showing an example of a lava-lithic bomb and associated bomb-sag. **b** Corresponding illustration showing the layer locations. The talus is shown in *dark gray*, the top of Rootless Tephra Sequence 1 (RTS1) is shown in *intermediate gray*, and larger clasts are depicted in *light gray*. Lower and upper bed-pair members for unit 1 are shown in *pale blue* and *dark blue*, respectively. Units 2.1.1 and 2.1.2 are shown in *pale green* and *dark green*, respectively. S layers are shown in *orange*

**Fig. 7:**
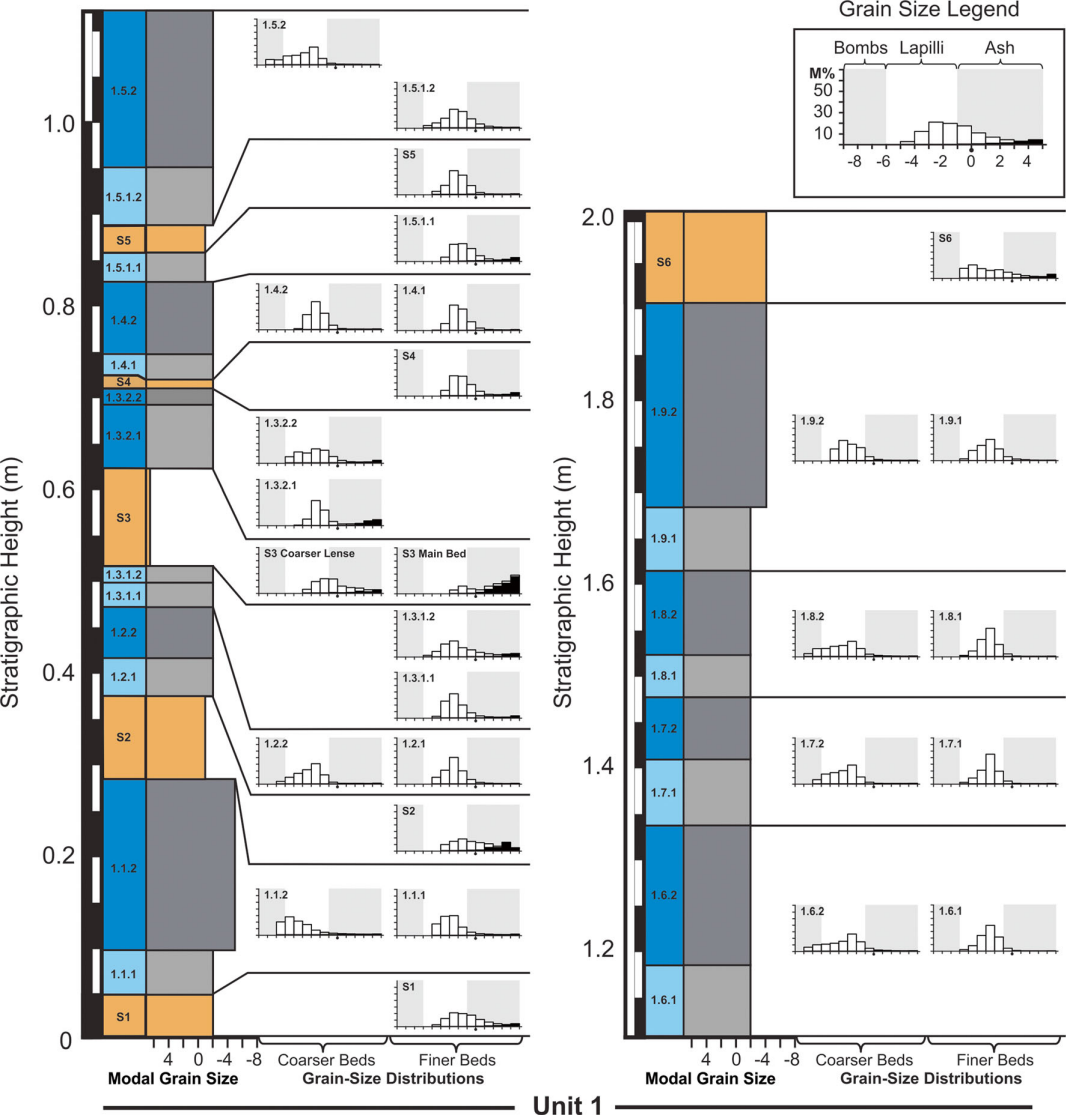
Detailed stratigraphic log for unit 1 showing the corresponding grain-size information for each layer. *Light* and *dark layers* represent lower and upper bed-pair members, respectively, whereas *orange units* represent S layers. In the histograms, *white columns* represent juvenile material, whereas *black columns* represent lacustrine sediment. The same conventions are applied in Fig. 8

Unit 1 is the lowermost unit in the stratigraphy, extending from 0 to 2.01 m (Figs. 3, 5, 6, and 7). Layers in this unit generally have a high abundance of lacustrine sediment and, relative to the other three units, unit 1 bed pairs tend to be thinner and finer grained. Unit 1 opens with S1, which is rich in diatomaceous lacustrine sediment (9%) and exhibits a distinctive ochre-orange color. This layer is 5–6 cm thick with a mean grain size of −1.1*ϕ* and poor sorting. S1 is overlain by unit 1.1.1, which is 5–6 cm thick with a mean grain size of 7.1*ϕ* and poor sorting. This layer represents the lower member of the first bed pair within RTS2 (i.e., unit 1.1). The upper member of the bed pair (unit 1.1.2) is 18–25 cm thick with a mean grain size of −4.6*ϕ* and poor sorting. Units 1.1.1 and 1.1.2 are separated by sharp contacts above and below, exhibit reverse grading, and are dominantly composed of juvenile material. However, unit 1.1 includes a substantially higher abundance of crystalline lava fragments (i.e., lava-lithics) than most of the other layers in the stratigraphy (i.e., 2–6% relative to a typical abundance of <1%). Unit 1.1.2 is overlain by S2, which is a 6–13-cm-thick layer with a mean grain size of 0.1*ϕ* and very poor sorting. This layer is particularly rich in lacustrine sediment (26%) and includes coarse ash lenses with a greater abundance of basaltic material than the majority of the matrix. S2 also exhibits low-angle cross-stratification and shows evidence of tephra interactions with local obstacles (e.g., bombs), including flow-aligned structures and asymmetrical deposition of material on the stoss and lee sides of the bombs protruding from the top of unit 1.1.2 (Fig. 6). These depositional patterns imply a unidirectional lateral flow of material from north to south. S2 also includes examples of basaltic bombs and lapilli that are embedded completely within the unit, and around these larger clasts, fine laminae within S2 are deformed (Fig. 6). The unit 1.2 bed pair is composed of a 3–5-cm-thick lower layer and a 4–6-cm-thick upper layer. Units 1.2.1 and 1.2.2 have mean grain sizes of −2.5*ϕ* and −3.2*ϕ*, respectively. Unit 1.3 is more complicated than the other bed pairs in this unit because there is a mud-rich layer, S3, interleaved between units 1.3.1 and 1.3.2. S3 contains the highest proportion of mud among any of the layers in unit 1 (60% lacustrine sediment abundance). S3 also exhibits gradational contacts above and below and appears to have mixed with the adjacent layers. Unit 1.3.1 is therefore subdivided into two: unit 1.3.1.1, which is a 3–4-cm-thick unmixed lower layer with a mean grain size of −2.3*ϕ*, low abundance of mud (1%), and poor sorting, and unit 1.3.1.2, which is a 2–4-cm-thick layer of finer-grained ($\overline{\sigma}$ = −1.7) material with a greater abundance of lacustrine sediment (10%) and very poor sorting. Similarly, unit 1.3.2 is subdivided into unit 1.3.2.1, which is a 6–8-cm-thick layer that is mixed with the top of S3, and unit 1.3.2.2, which is a 2–3-cm-thick layer of coarse-grained tephra that appears unmixed with S3. Unit 1.3.2.1 has a mean grain size of −1.2*ϕ*, whereas unit 1.3.2.2 has a mean grain size of −2.7*ϕ*. Both layers are very poorly sorted (${\overline{\sigma}}_{\phi }$ = 3.4 for unit 1.3.2.1 versus ${\overline{\sigma}}_{\phi }$ = 2.2 for unit 1.3.2.2). Unit 1.3 is overlain by a 1-cm-thick mud-rich layer (S4), which exhibits only slight variations in thickness. Unit 1.4 is a reversely graded bed pair with a 3-cm-thick lower fine-grained (*ϕ* = 1.9) layer (unit 1.4.1) and an 8–9-cm-thick coarser-grained (${\overline{\sigma}}_{\phi }$ = −2.4) upper component. Unit 1.5 represents a change toward thicker bed pairs in unit 1; however, the lower member of the bed pair (unit 1.5.1) is mixed with a 3-cm-thick S layer, S5, which has a gradational lower contact and a sharp upper contact. This implies that S5 was emplaced concurrently with the opening phases of unit 1.5.1. The lower mixed component of unit 1.5.1 (i.e., unit 1.5.1.1) has ${\overline{\sigma}}_{\phi }$ = −1.2 and contains 11% mud, whereas the unmixed upper part of unit 1.5.1 (i.e., unit 1.5.1.2) has a slightly coarser grain-size distribution and much lower mud (${\overline{\sigma}}_{\phi }$ = −2.1 and <1% mud). Unit 1.5.2 is a 16–19-cm-thick coarse-grained layer (${\overline{\sigma}}_{\phi }$ = −3.9) that does not appear to have mixed at all with S5. Units 1.6, 1.7, 1.8, and 1.9 represent a succession of very similar reversely graded bed pairs each ranging in total thickness from 14 to 29 cm. The lower members of the bed pairs range in mean thickness from 5 to 7 cm, and the upper members range in mean thickness from 7 to 23 cm—with the thickest of the deposit in this succession being unit 1.9, which has a 5–7-cm-thick lower component (unit 1.9.1) and a 21–29-cm-thick upper component. These four bed pairs are dominantly composed of basaltic tephra with a minor component of mud. There are no mud-rich S layers interrupting the sequence. Unit 1.9 is overlain by another mud-rich layer (S6), which is 10–13 cm thick. This deposit may mark either the last S layer in unit 1 or the first layer of the unit 2 sequence, but given the high concentrations of mud-rich layers in unit 1 and the lack of a gradational boundary at the top of S6, we have assigned S6 to the top of unit 1. S6 exhibits small variations where the deposit pinches and swells in thickness in response to the local topography, and like the other S layers, it is more orange in color and exhibits fine internal laminations and low-angle cross-stratification.

#### Unit 2

**Fig. 8:**
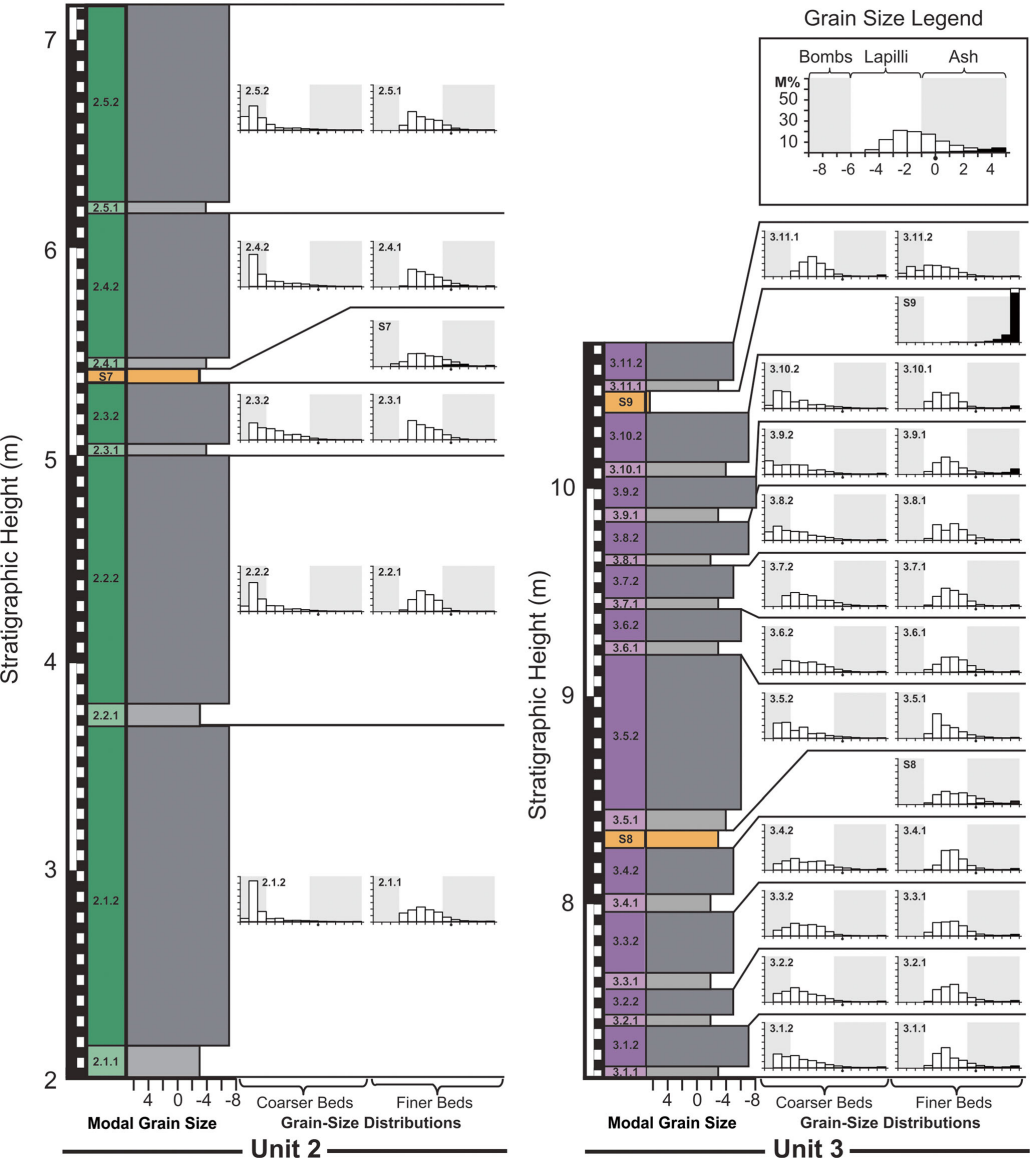
Detailed stratigraphic log for units 2 and 3 showing the corresponding grain-size information for each layer. Note the change in vertical scale relative to Fig. 7

Unit 2 is the thickest unit in the tephra sequence, spanning from 2.01 to 7.16 m in the stratigraphy (Figs. 3, 5, and 8). It is characterized by bed pairs that are generally thicker and more coarse grained than other bed pairs within the stratigraphy. Modal and mean grain sizes among each of the bed-pair members in unit 2 are relatively consistent. Specifically, lower bed-pair layers have a mean grain size of −3.0*ϕ*, with a range from −2.6*ϕ* to −3.1*ϕ*, and upper bed-pair layers have a mean grain size of −6.3*ϕ*, with a range from −5.3*ϕ* to −7.0*ϕ*. However, the average sorting for the layers tends to decrease slightly with increasing stratigraphic height for the fine bed-pair layers, from ${\overline{\sigma}}_{\phi }$ = 1.8 to ${\overline{\sigma}}_{\phi }$ = 1.6, and it slightly increases for the coarse bed-pair layers, from ${\overline{\sigma}}_{\phi }$ = 1.3 to ${\overline{\sigma}}_{\phi }$ = 1.8. Overall, there are five reversely graded bed pairs within units 2.1, 2.2, 2.3, 2.4, and 2.5, and one mud-rich layer (S7), which occurs between units 2.3.2 and 2.4.1. Each of the bed pairs has sharp contacts above and below and is dominantly composed of basaltic tephra. Unit 2.1.1 is a 13–18-cm-thick fine-grained lower bed-pair member located at the base of unit 2. This layer is directly overlain by unit 2.1.2, which is the thickest layer in the entire tephra succession, ranging in thickness from 130 to 160 cm. Units 2.1.1 and 2.1.2 have mean grain sizes of −3.1*ϕ* and −7.0*ϕ*, respectively, and are discussed in more detail in the context of lateral thickness variations (“Lateral facies variation”). Unit 2.2.1 is 9–14-cm-thick and unit 2.2.2 is 120–130-cm-thick. These layers are very similar in their structure to units 2.1.1 and 2.1.2, respectively. Unit 2.3 marks a change to considerably thinner bed pairs within the unit—with unit 2.3.1 being 5–12-cm-thick and unit 2.3.2 being 30–40-cm-thick. Unit 2.3.1 has a mean grain size of −3.0*ϕ* and unit 2.3.1 has a mean grain size of −5.3*ϕ*. Unit 2.3.2 is directly overlain by S7, which is a mud-rich layer with pinch-and-swell layer geometry, and sharp contacts above and below. S7 is 5–8-cm-thick and includes fine laminae and low-angle cross-stratification. Unit 2.4.1 is another fine-grained lower bed-pair member that is 5–8-cm-thick, which has the highest mud content of any layer within unit 2 (nearly 5%). Unit 2.4.1 is directly overlain by a 60–70-cm-thick coarse-grained upper bed-pair layer (unit 2.4.2), with a mud abundance of <1%. Low mud contents are typical of most bed-pair layers in this unit. Unit 2.5 is the uppermost bed pair in unit 2, and it opens with a fine-grained layer (unit 2.5.1) that is 5–7 cm thick and the second-most mud-rich (3–4% mud content) of the bed-pair layers within unit 2. This is overlain by unit 2.5.2, which is a 90–95-cm-thick coarse-grained upper bed pair.

#### Unit 3

Unit 3 ranges in stratigraphic height from 7.16 to 10.70 m (Figs. 3, 5, and 8), and like unit 2, it is dominantly composed of a series of bed pairs. However, relative to unit 2, unit 3 includes much thinner bed pairs. For instance, unit 2 includes five lower and upper bed-pair members with mean thicknesses of 7.6 and 94.0 cm, respectively, whereas unit 3 includes 11 lower and upper bed-pair layers with mean thicknesses of 6.2 and 24.4 cm, respectively. Among the unit 3 bed pairs, most of the lower layers range in thickness from 5 to 12 cm, whereas the upper bed-pair layers are 12–35 cm thick, the exception being unit 3.5.2, which is 70–80 cm thick. In terms of grain size, the lower bed-pair layers have a mean grain size of −2.9*ϕ*, ranging from ${\overline{\sigma}}_{\phi }$ = −2.5 to −3.4, and the coarse-grained bed pairs have ${\overline{\sigma}}_{\phi }$ = −4.9, with a range of ${\overline{\sigma}}_{\phi }$ = −4.0 to −5.7. Thus, relative to unit 2, the key characteristics of unit 3 are that it is composed of both thinner and finer-grained layers. Unit 3 layers also have higher abundances of mud than the layers in unit 2, with a mean layer abundance of 7% for unit 3 versus 2% for unit 2 (this average includes contributions from S layers). Unit 3 includes also two S layers, whereas unit 2 has only one. The unit 3 S layers include the 7–9-cm-thick S8 layer and the 7–11-cm-thick S9 layer. Both S8 and S9 exhibit sharp contacts above and below and were emplaced between units 3.4 and 3.5, and units 3.9 and 3.10, respectively. Among all of the S layers in the tephra sequence, S9 includes the highest proportion of lacustrine sediment (91%).

#### Unit 4

Unit 4 is the final unit in the tephra sequence, extending from 10.70 to 13.55 m (Figs. 3 and 5). This unit is characterized by three alternating fine- and coarse-grained bed pairs that become increasingly welded with stratigraphic height, grading into a welded “spatter cap” at the top of the succession. During quarrying operations, unconsolidated tephra deposited on top of unit 4 was removed, but comparisons with adjacent parts of the stratigraphy and historical records by von Komorowicz (1912) suggest that unit 4 is well preserved at this locality. Unit 4.1 is composed of two parts, unit 4.1.1, which is an 8–13-cm-thick fine-grained (${\overline{\sigma}}_{\phi }$ = −3.3) bed-pair layer, and unit 4.1.2, which is a 30–35-cm-thick coarse-grained bed-pair layer (grain-size data are not available for this layer due to tack welding). Unit 4.2 is similarly composed of an 8–12-cm-thick fine-grained ${\overline{\sigma}}_{\phi }$ = −3.3) basal layer (unit 4.2.1), which is overlain by a 30–50-cm-thick upper bed-pair layer (unit 4.2.2; again, grain-size data are not available for this layer due to welding, intermediate between incipient and moderate welding). Unit 4.3 is the uppermost unit in the tephra sequence, and both the lower 30–35-cm-thick layer (unit 4.3.1) and the upper 140–170-cm-thick layer (unit 4.3.2) exhibit moderate to dense welding. Unit 4 does not include any mud-rich S layers. Welded layers of this unit do not exhibit evidence of post-depositional lateral flow (i.e., rheomorphic, or clastogenic flow), but other large rootless cones within Rauðhólar do include welded spatter caps that exhibit evidence of rheomorphic flow. Evidence of rheomorphic flow typically includes dissolution of clast boundaries through melting and assimilation of their exteriors combined with down-slope deformation of the material, shearing and elongation of bubbles into flow parallel chains of oblate vesicles, and an overall increase in the density of the deposit.

### Lateral facies variation

To quantify and compare layer-thinning relationships within lower and upper bed-pair members, units 2.1.1 and 2.1.2 were chosen as archetypes because of their representative structure and accessibility within the outcrop. For these two layers, we measured the layer thicknesses at 1-m intervals along the southern cross section.

**Fig. 9:**
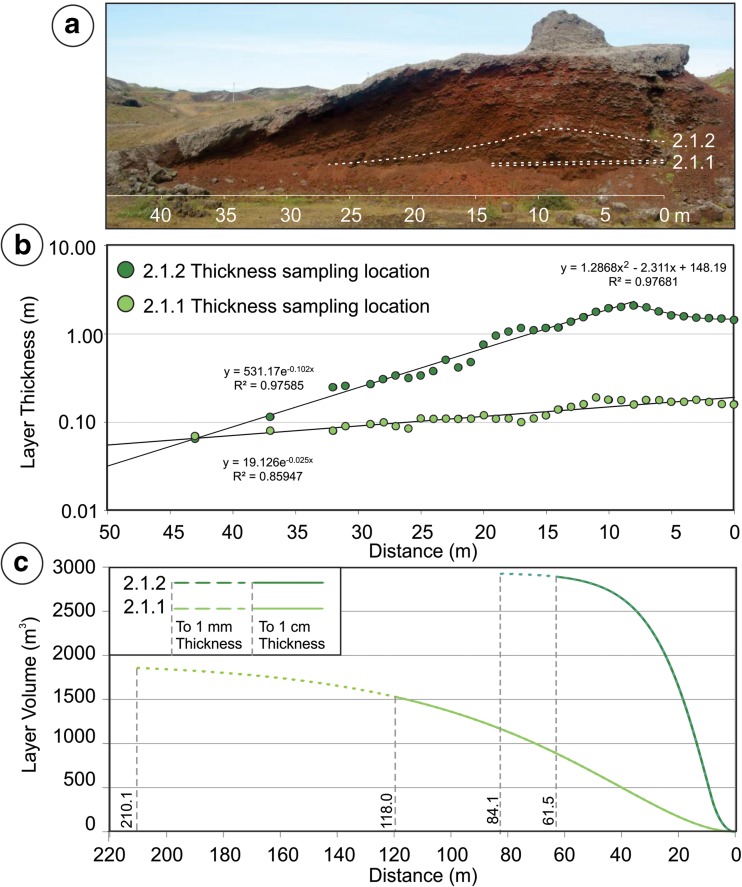
**a** Locations of layers 2.1.1 and 2.1.2. Lateral variations in thickness for layers 2.1 and unit 2.2. These layers are partially buried by talus but were excavated to obtain the thickness measurements shown in **b** *.* The thickness variations within layer 2.1.1 are well described by a single exponential function, whereas layer 2.1.2 is fit by a second-order polynomial in the region from 0 to 8 m and exponential function at distances ≥8 m. **c** Using the best-fit equations from **b**, volumes of solids of revolution were calculated for both layers. Limits for the integrations were obtained by calculating the distance required for each layer to thin to 1 cm (*solid lines*) and to 1 mm (*dashed lines*)

The southern quarry exposure approximates a radial transect away from the tephra source region for RTS2, but it is important to note that the cross section may be oblique with respect to a true radial profile away from the vent. Thus, apparent thinning geometries provide relative information about the thickness variations of the layers within the deposit but cannot be used directly to calculate absolute thinning half distances (Pyle 1989). Nonetheless, thickness variations for units 2.1.1 and 2.1.2 clearly demonstrate that lower bed-pair members thin more gradually than the upper bed-pair members (Fig. 9), which implies that lower bed-pair layers have a more sheet-like geometry, whereas upper bed pairs have a more conical shape. Unit 2.1.1 is well described (*R* ^2^ = 0.859) by an exponential thinning relationship, *y* = 19.126e^−0.025*x*^, where *x* is the distance measured in meters away from the inferred source region and *y* is the thickness of the deposit measured in centimeters. This yields an “apparent” thinning half distance of 27.73 m. In contrast, for distances greater than *x* = 8 m, unit 2.1.2 is well described (*R* ^2^ = 0.976) by an exponential thinning relationship of *y* = 531.17e^−0.102*x*^, which corresponds to an “apparent” thinning half distance of 6.80 m. However, in the region from *x* = 0 to 8 m, the thickness of the deposit significantly departs from an exponential function (Fig. 9) and is better described by a second-order polynomial (*R* ^2^ = 0.977), *y* = 1.2868*x* ^2^ − 2.311*x* + 148.19. This suggests that the region from *x* = 0 to 8 m may be part of the crater facies, rather than an outer cone facies.

Assuming radial symmetry about the vent location, we estimate the volume of solid revolution, *V*, for units 2.1.1 and 2.1.2 using shell integration. For the curve *f*(*x*), which describes the thickness variation within a given layer, *V* is obtained by calculating the area beneath the curve *f*(*x*), between the lines *x* = *a* and *x* = *b*, and rotating it about the *y*-axis (centered on the vent location),

2 $$ V=2\;\pi\;{\displaystyle \underset{a}{\overset{b}{\int }}x\;\left|\;f\right.}(x)\;\left|\;dx\right. $$

These calculations assume radial symmetry about a rootless vent located at *x* = 0 m, but given the uncertainty in this condition, volume estimates for units 2.1.1 and 2.1.2 are most appropriately considered on a relative basis to one another, rather than as a measure of true volume. To calculate the volume of unit 2.1.1, we integrate between *a* = 0 m and *b* = 118.05 m, where *b* corresponds to the distance where the deposit is expected to thin to 1 cm. Changing the boundary to the distance that the deposit would thin to 1 mm (*b* = 210.15 m) increases the estimated volume of the deposit from 1525.9 to 1859.9 m^3^ (a difference of 21%). However, such a thin layer would be composed of very fine particles that would be strongly affected by turbulence, which could affect the continuity of the distal tephra deposits and make the material very difficult to discern from the products of other nearby rootless eruption sites. Nonetheless, the distal component of the lower bed-pair layers is volumetrically significant and likely a major constituent of the tephra platform upon which the VRCs are constructed. To estimate the volume of unit 2.1.2, we use the second-order polynomial for *f*(*x*) between *a* = 0 m and *b* = 8 m, which yields a volume of 356.0 m^3^. To this, we add the volume of revolution obtained for the exponential function, evaluated between *a* = 8 m and *b* = 61.52 m. This value of *b* corresponds to the distance where the layer would be expected to thin to 1 cm, but if we were to extend this range to 1 mm (*b* = 118.05 m), the estimated total volume of the deposit would only increase from 2896.9 to 2926.2 m^3^ (a 1% difference). Thus, given the rapid thinning of unit 2.1.2, material from upper bed-pair layers is unlikely to be a major contributor to the distal platform facies because most of the tephra would accumulate around the rootless vent—either providing infill for the crater facies or constructing the outer cone facies. The results show that although unit 2.1.2 is ~12 times thicker than unit 2.1.1 in the proximal tephra stratigraphy (155 versus 13 cm modal thickness), the upper unit is only 1.57 to 1.89 times more voluminous, depending on whether *b* is evaluated using the 1-mm or 1-cm thickness threshold, respectively. Therefore, while lower bed-pair members may appear thin within the proximal stratigraphy, they have a sheet-like geometry, which implies widespread distribution. Consequently, lower bed pairs represent a critical part of the overall structure of VRC groups and may be the dominant contributors to the VRC sheet facies, while upper bed-pair layers represent the dominant component of the VRC cone facies—with both layers contributing to the construction of the VRC platform facies (Hamilton et al. 2010a).

## Interpretation

### The origin of bed-pair layering

The stratigraphy is dominantly composed of a rhythmic succession of bed pairs, which we interpret to be fall deposits generated by discrete episodes of explosive activity from the RTS2 rootless eruption site within Cone 53. Both the lower and upper bed-pair members exhibit conformal layering that drapes the underlying topography, and they are either massive or reversely graded. This is consistent with a depositional mechanism involving tephra fall. The lower bed-pair layers also include a contribution of ballistics, including lava-lithic blocks and spatter clasts that deformed on impact; however, spatter clasts are common within the upper bed-pair layers. The lower member of each bed pair is interpreted to be the energetic opening phase of an explosion cycle, as evidenced by its finer grain size and greater dispersal (i.e., a greater thinning half distance) of the deposits relative to the upper bed-pair layer. The lower bed-pair members have more of a sheet-like geometry, evidenced by their greater thinning half distances, than the upper bed-pair members, which are more conically shaped and have a shorter thinning half distance. We therefore interpret the upper bed-pair layers to be the products of a lower-energy ballistic phase at the end of each explosion cycle, likely including spray-like jets or lava fountains as well as the products of discrete bubble burst events (Mattox and Mangan 1997; Greeley and Fagents 2001), with bubble burst explosions becoming increasingly common in units 2–4. The repetitive nature of the bed pairs, separated by sharp contacts above and below, implies that each explosion cycle occurred as a discrete event, separated by a period of repose. The paired layering structure within RTS2 also suggests that the bed pairs were formed during a two-stage process, not as a result of series of explosions that each produced a single uprush of material.

One possibility that could account for the paired layering is a process that dynamically segregates material from the rootless explosion site and tephra derived from the overlying lava column. A similar process was described by Andrews et al. (2014), who used laboratory explosion experiments in granular material to show that debris jet deposits can be formed by a two-stage process involving cavitation and subsequent granular fountaining. During the first stage, a slug of rapidly expanding gas pierces the overlying material and shoots through the overburden to the surface to form an incipient rootless conduit. During the second stage, a transient cavity—formed by the passage of the gas through the overburden—will continue to expand, collapse, and rebound to produce a second fountaining event. These experiments involved granular material, rather than multi-phase lava overlying a layer of sedimentary material, but there may be some similarities in terms of an initially energetic slug of gas and tephra rising from the rootless eruption site and piercing through the overlying lava column to deposit material onto the surface of the lava before a second, lower-energy lava fountaining stage is initiated. This would result in a paired layering structure with more deeply sourced material being deposited in the lower layer and more shallowly derived material deposited in the upper layer. The lower layer, resulting from a more highly energetic opening explosion phase, would also be expected to have a finer grain-size distribution and wider dispersal than the upper layer, which would have been produced during a weaker lava fountaining-like phase.

We infer that each explosion cycle began after a period of repose in which groundwater recharged into the rootless eruption site. This enabled a hydrodynamic premixing phase in which fluidal lava and adjacent wet sediments were able to mingle intimately under non-explosive (i.e., stable vapor film) conditions. Material that consists of fluidal lava with embedded mud clots (e.g., Fig. 4f) is interpreted to have been preserved at this MFCI stage. The core of the Elliðaá lava flow is not exposed in the vicinity of Rauðhólar, but Reynolds et al. (2015) describe cross sections through an analogous rootless cone group in the Ice Harbor flow field of the Columbia River Basalt Province, which reveals the presence of funnel-shaped, upward flaring features in the lava that include spatter and irregularly shaped cavities. Reynolds et al. (2015) interpret these structures to be partially infilled rootless conduits, which is in accord with the transient conduit formation mechanisms proposed here.

Hydrodynamic premixing within the rootless eruption site would have continued until a triggering mechanism (e.g., undercooling, rapid condensation, and vapor film collapse, or propagation of a pressure pulse through the mixture) initiated fine fragmentation of the lava and wet sediment mixture. Water within the system then underwent rapid vaporization and runaway expansion. The resulting MFCI explosion generated a highly energetic slug of material that expanded outwards and pierced through the overlying lava column to deposit a widely dispersed layer of fine-grained material, which included both juvenile material and lacustrine sediment. Passage of the high-energy slug through the overlying lava column would also cause the formation and collapse of a transient cavity, which in turn would have caused the molten lava to rebound and deposit spatter-rich material near the rootless vent. The collapse of the transient cavity may also have helped to initiate a subsequent stage of lava fountaining by forcing molten lava downward and into the rootless eruption site, which would help to drive continued dynamic mixing of lava and underlying wet sediments. Both of these processes—rebounding of the collapsed transient cavity and enhanced dynamic mixing and the generation of new MFCI explosions—would have helped to construct the remainder of the upper bed-pair member. However, these MFCI explosions would have been less energetic than the initial explosion because partial excavation of lava above the rootless eruption site would have enabled the system to reach the explosive decompression stage at an earlier point due to the lower confining pressure of the overlying lava column (see Eq. 1).

The overall division of rootless tephra stratigraphy into fine- and coarse-grained bed pairs raises important questions regarding the efficiency of rootless MFCI explosions and the role of “active” versus “passive” particles in the process. In “Molten fuel–coolant interaction theory,” we described how ideally efficient MFCI explosions require the development of uniform melt fragments with a diameter of approximately 1 μm (Wohletz 1986); however, the mean grain size ranges from fine to coarse lapilli among the lower bed-pair layers and from coarse lapilli to bomb-sized clasts among the upper bed pairs. This suggests that rootless MFCIs are far from being ideally efficient; the majority of the tephra generated from a rootless explosion, particularly in the upper bed-pair layers, may not have been directly involved with the MFCI, but rather was ejected from the upper lava column as a consequence of underlying explosions. Fitch et al. (2017) explores this theme in more detail by investigating the fragmentation mechanisms associated with rootless explosions at Rauðhólar.

In terms of understanding the relationship between lava–substrate mixing environments and explosion type, Mattox and Mangan (1997) described four general types of littoral hydrovolcanic explosion including tephra jets, lithic blasts, bubble bursts, and littoral lava fountains. Mattox and Mangan (1997) attributed tephra jets and lithic blast events to open mixing processes, which begin with bench collapse events that expose an active lava tube or incandescent rock scarp to wave action, thereby forcing coarse mixing of seawater and lava. In contrast, they attribute bubble bursts and littoral lava fountaining to confined mixing, which occurs when a lava tube is situated at, or below, sea level, and allows water to enter into the tube system though fractures in the lava. Within this context, bed-pair deposits within RTS2 are generally consistent with the products of lava fountaining and bubble burst events generated by MFCI explosions under confined mixing conditions, rather than open mixing conditions. This agrees with the inferred lacustrine paleo-environment for Rauðhólar, which would have been less dynamic than a marine littoral setting. Pāhoehoe emplacement under stable conditions would also have favored the development of well-established internal lava pathways (i.e., lava tubes), particularly in the deeper parts of the lake basin. Lava tube formation may therefore have been a critical precondition for enabling efficient resupply of lava to the rootless eruption sites as they developed, and without this mechanism of lava replenishment, the rootless eruptions would have been lava limited, rather than water limited, and failed to construct such large edifices.

In the presence of excess lava, depletion of groundwater in the vicinity of the rootless eruption site would have lowered MFCI efficiency as the water-to-melt mass ratio decreased. The process would have continued until the ratio descended below a critical value required to trigger the next MFCI explosion. At this point, the rootless eruption would have ceased until groundwater infiltrated back into system and enabled the development of another explosive cycle and the production of an additional bed pair in the stratigraphy. In the context of RTS2, this process was repeated a total of 28 times to construct the 28 bed pairs represented by units 1–4. The overall increase in grain size with stratigraphic height, combined with the occurrence of welded deposits near the top of unit 4, supports the conclusion that there was a gradual decrease in the efficiency of the MFCI explosions with time. This may have been due to the depletion of available coolant (i.e., groundwater) through a drawdown of the local piezometric surface below the depth of the rootless eruption site. Alternatively, the reduction in available water may have been due to the depletion of wet sediments in the rootless eruption site, which may have only been a few meters thick based on nearby drill core records (Tómasson et al. 1977). However, other processes such as changes in the rootless conduit/vent geometry, mixing conditions, lava temperatures, and coolant impurities may have also contributed to variations in MFCI efficiency (White 1996; Wohletz 2002).

### The origin of the S layers

The stratigraphy also includes nine S layers, which are mud-rich deposits that interrupt the regular sequence of alternating fine- and coarse-grained bed-pair layering. S layers typically occur as isolated layers with sharp contacts above and below; however, in unit 1, S3 and S5 are mixed with units 1.3.2 and 1.5.1, respectively. This implies that S layer emplacement does not follow a regular pattern and that the S layer may either be emplaced between the deposition of successive bed-pair units, or concurrently with them. S layers generally include a high abundance of lacustrine sediment and show evidence of fine internal lamination, low-angle cross-stratification, and pinch-and-swell layer geometries that vary in response to the local topography. Additionally, the S layers show systematic differences in the deposition on the lee and stoss sides of high-standing obstacles, which together with cross-stratification implies lateral flow. Where present within the S layers, coarse lapilli and bombs appear to have deformed the internal layering structure in a process consistent with the formation of bomb-sags within deformable (typically wet) sediments (Lorenz 1973, 2007; Schmincke 2004). High concentrations of condensable water associated with these explosions (Wohletz 2002) may have favored gravitational collapse of the ejecta. We therefore interpret the S-layer deposits to result from dilute, laterally moving pyroclastic density currents (i.e., surges) similar to those documented by Belousov et al. (2011) in association with explosions generated by the flow of lava over snow and ice and by Reynolds et al. (2015) in the context of rootless explosions occurring in a lacustrine setting.

Unit 1 opens with an S layer, which implies that the initiation of a new rootless eruption site coincided with the emplacement of a dilute pyroclastic density current that was rich in lacustrine sediment and condensable water. Unit 1 also includes six of the nine S layers, two of which are mixed with bed-pair layers, which suggests that surges were the most frequent during the opening phases of the new eruption and that some surges were emplaced concurrently with bed pairs formed by tephra fall and ballistic emplacement of ejecta. All of these factors support the conclusion that the opening phase of the eruption (i.e., unit 1) occurred under conditions with ample groundwater availability and that the gradual decrease in the frequency of surge-forming explosions during the later stages of cone building was related to a gradual desiccation of the rootless eruption site due to the rate of water extraction exceeding the rate of water recharge, with recharge being limited by pore water flow velocities through the substrate and/or the development of physical blockages associated with the lava and associated rootless explosions.

## Conclusions

Quarry exposures within Rauðhólar provide extraordinary cross sections through the stratigraphy of numerous rootless cones. Among these, the 13.55-m-thick vent proximal section through Cone 53 provides the best example of a complete rootless tephra sequence (RTS). This section (RTS2) divides into four units. Unit 1 represents the opening phase of a new rootless eruption site and includes dilute pyroclastic density current (i.e., surge) deposits and lapilli-dominated bed pairs, composed of a fine-grained basal layer and a coarser-grained upper layer, which we infer were emplaced through a combination of tephra fall from a weak ash-plume and ballistic ejecta from lava fountains and/or bubble burst events. Based on the grain-size and thinning half-distance relationships, we infer that the explosions associated with unit 1 were the most energetic within the sequence and were driven by relatively efficient MFCI explosions with higher conversion ratios (CR; Eq. 1) than the other units in the tephra sequence. Units 2 and 3 are dominated by a rhythmic succession of bed pairs, with unit 2 deposits being thicker and the most coarsely grained, whereas unit 3 layers are more numerous and generally much thinner and finer grained. Unit 3 also contains a higher abundance of lacustrine sediment, but relative to unit 1 the mud clots in unit 3 appear more desiccated. Unit 4 includes a series of bed-pair units that exhibit increasing degrees of welding near the top of the section. Welding may be explained by a decreased MFCI efficiency, which would reduce the conversion of thermal energy into mechanical energy (i.e., fragmentation) and kinetic energy (i.e., dispersal) of the ejecta. This would result in the eruption of material that was less thoroughly fragmented (i.e., coarser) and ejected with lower velocities, which would promote the rapid accumulation and welding of hot tephra deposits near the rootless vent.

Overall, the observed tephra succession is consistent with an explosive MFCI system in which the water-to-melt ratio gradually decreased with time from a highly efficient state, during the emplacement of unit 1, to a less efficient state, during the deposition of unit 4. This pattern is interpreted in the context of local depletion of coolant (i.e., groundwater or wet sediments) in the presence of excess fuel (i.e., molten lava). However, groundwater recharge may have temporarily re-primed the MFCI system and enabled renewed cycles of vigorous explosive activity after periods of repose. Thus, while groundwater depletion would result in an overall decrease in MFCI efficiency with time, each unit is composed of a series of energetic explosive cycles that could reflect temporary increases in MFCI efficiency due to groundwater recharge. However, this process was complicated by the emplacement of a series of surge deposits that may have been associated with initiation of new explosion sites near the main rootless eruption site. These new explosions may have accessed previously undisrupted pockets of wet sedimentary material, but the prevalence of juvenile (i.e., basaltic) material within each of the S layers suggests that they were generated by MFCI explosions involving an intimate mixture of substrate sediment and lava, rather than being the product of pure steam (i.e., phreatic) explosions.

This study demonstrates that VRCs are complex structures, which can be composed of multiple rootless tephra sequences originating from nearby, but distinct sources. Rootless tephra deposits can include contributions from fall emplacement, ballistic ejecta, and pyroclastic density currents. However, the bulk of a rootless cone’s stratigraphy is dominated by a rhythmic series of bed pairs, which imply cycles of explosive activity. Relative to the layers that compose the upper component of each bed pair, lower bed pairs are typically finer grained, more widely dispersed, and include twice as much lacustrine sediment. This suggests that these layers result from more intense (i.e., higher efficiency) explosions involving a larger proportion of wet sediment. However, the prevalence of lapilli in the lower bed pair layers, and lapilli to bomb-sized material in the upper bed pairs, implies that very little molten lava directly participated in highly efficient thermodynamic interactions that would be analogous to the role of “active” particles in laboratory MFCI experiments. This means that heat transfer from only a small proportion of the material involved in a rootless eruption is actually responsible for supplying the energy needed to drive the explosions. In terms of morphology, lower bed pairs are the dominant contributor to the distal sheet facies; the upper bed pairs are the main contributor to the vent-proximal cone facies, and both layer types contribute to the vent-proximal to medial platform facies. These observations contribute to an improved understanding of the hazards associated with rootless eruptions and their morphological expression in the geologic record by (1) better documenting the range of eruptive products generated by rootless eruptions; (2) assessing their similarities and differences relative to laboratory MFCI experiments; and (3) characterizing the facies-associated VRCs and their morphologies, which is important for identifying the products of explosive lava–water interactions on Earth and other planetary surfaces.

## Electronic supplementary material

ESM 1 (XLSX 1.19 mb).

ESM 2 (PDF 472 kb).

## References

[CR1] Andrews B (2003) Eruptive and depositional mechanisms of an Eocene shallow submarine volcano, Moeraki Peninsula, New Zealand, in explosive subaqueous volcanism (eds. JDL White, JL Smellie, and DA Clague), American Geophysical Union, Washington, DC, doi:10.1029/140GM11

[CR2] Andrews RG, White JDL, Düring T, Zimanowski B (2014). Discrete blasts in granular material yield two-stage process of cavitation and granular fountaining. Geophys Res Lett.

[CR3] Belousov A, Behncke B, Belousova M (2011). Generation of pyroclastic flows by explosive interaction of lava flows with ice/water-saturated substrate. J Volcanol Geotherm Res.

[CR4] Blott SJ, Pye K (2001). GRADISTAT: a grain size distribution and statistics package for the analysis of unconsolidated sediments. Earth Surf Process Landf.

[CR5] Büttner R, Dellino P, La Volpe L, Lorenz V, Zimanowski B (2002). Thermohydraulic explosions in phreatomagmatic eruptions as evidenced by the comparison between pyroclasts and products from molten fuel coolant interaction experiments. J Geophys Res.

[CR6] Colgate SA, Sigurgeirsson T (1973). Dynamic mixing of water and lava. Nature.

[CR7] Drumheller DS (1979). The initiation of melt fragmentation in fuel-coolant interactions. Nucl Sci Eng.

[CR8] Fagents SA, Thordarson T, Chapman MG (2007). Rootless volcanic cones in Iceland and on Mars. The geology of Mars: evidence from earth-based analogs.

[CR9] Fagents SA, Lanagan P, Greeley R (2002) Rootless cones on Mars: a consequence of lava–ground ice interaction. In: Smellie JL, chapman MG (eds) Volcano-ice interaction on earth and Mars. Geol Soc Spec Publ 202:295–317

[CR10] Fisher RV (1968). Puu Hou littoral cones, Hawaii. Geol Rundsch.

[CR11] Fitch EP, Fagents SA, Thordarson T, Hamilton CW (2017) Fragmentation mechanisms associated with explosive lava–water interactions in a lacustrine environment. Bull Volcanol. doi:10.1007/s00445-016-1087-3

[CR12] Frey H, Jarosewich M (1982). Subkilometer Martian volcanoes: properties and possible terrestrial analogs. J Geophys Res.

[CR13] Frey H, Lowry BL, Chase SA (1979). Pseudocraters on Mars. J Geophys Res.

[CR14] Greeley R, Fagents SA (2001). Icelandic pseudocraters as analogs to some volcanic cones on Mars. J Geophys Res.

[CR15] Guðmundsson MT, Sigmundsson F, Björnsson H (1997). Ice–volcano interaction of the 1996 Gjálp subglacial eruption, Vatnajökull, Iceland. Nature.

[CR16] Hamilton CW, Thordarson T, Fagents SA (2010a) Explosive lava–water interactions I: architecture and emplacement chronology of volcanic rootless cone groups in the 1783–1784 Laki lava flow. Bull Volcanol 72(4):449–467. doi:10.1007/s00445-009-0330-6

[CR17] Hamilton CW, Fagents SA, Thordarson T (2010b) Explosive lava–water interaction II: self-organization processes among volcanic rootless eruption sites in the 1783–1784 Laki lava flow, Iceland. Bull of Volcanol 72(4):469–485. doi:10.1007/s00445-009-0331-5

[CR18] Hamilton CW, Fagents SA, Wilson L (2010). Explosive lava–water interactions in Elysium Planitia, Mars: constraints on the formation of the Tartarus Colles cone groups. J Geophys Res—Planets.

[CR19] Hamilton CW, Fagents SA, Thordarson T (2011). Lava–ground ice interactions in Elysium Planitia, Mars: geomorphological and geospatial analysis of the Tartarus Colles cone groups. J Geophys Res—Planets.

[CR20] Hamilton CW, Glaze LS, James MR, Baloga SM (2013). Topographic and stochastic influences on pāhoehoe lava lobe emplacement. Bull Volcanol.

[CR21] Hamilton CW, Moersch JE, Scheidt SP (2015) Applications of unmanned aerial vehicles to the study of volcanic landforms. GSA Annual Meeting, Baltimore, MD, USA. Abstract 264973. Poster

[CR22] Head JW, Wilson L (2002) Mars: a review and synthesis of general environments and geological settings of magma/H_2_O interactions. In: Smellie JL, Chapman MG (eds) Volcano-ice interaction on Earth and Mars. Geol Soc Lond Spec Publ 202:27–57

[CR23] Hickson CJ (2000). Physical controls and resulting morphological forms of Quaternary ice-contact volcanoes in western Canada. Geomorphology.

[CR24] Hon K, Kauahikaua J, Denlinger R, MacKay R (1994). Emplacement and inflation of pahoehoe sheet flows: observations and measurements of active lava flows on Kilauea Volcano, Hawaii. Geol Soc Am Bull.

[CR25] Jaeger WL, Keszthelyi LP, McEwen LP, Dundas CM, Russell PS (2007) Athabasca Valles, Mars: a lava-draped channel system. Science 317(5845):1709–1711. doi:10.1126/science.1143315 10.1126/science.1143315 17885126

[CR26] Jurado-Chichay Z, Rowland SK, Walker GPL (1996). The formation of circular littoral cones from tube-fed pahoehoe: Mauna Loa, Hawaii. Bull Volcanol.

[CR27] Kokelaar P (1986) Magma–water interactions in subaqueous and emergent basaltic. Bull Volcanol 48(5):275–289. doi:10.1007/BF01081756

[CR28] Lanagan PD, McEwen AS, Keszthelyi LP, Thordarson T (2001). Rootless cones on Mars indicating the presence of shallow equatorial ground ice in recent times. Geophys Res Lett.

[CR29] Lescinsky DT, Fink JH (2000) Lava and ice interaction at stratovolcanoes: use of characteristic features to determine past glacial extents and future volcanic hazards. J Geophys Res: Solid Earth 105(B10):23711–23726

[CR30] Lorenz V (1973). On the formation of maars. Bull Volcanol.

[CR31] Lorenz V (2007). Syn- and posteruptive hazards of maar-diatreme volcanoes. J Volcanol Geotherm Res.

[CR32] Mattox TN, Mangan MT (1997). Littoral hydrovolcanic explosions: a case study of lava-seawater interaction at Kilauea Volcano. J Volcanol Geotherm Res.

[CR33] Moore JG, Ault WU (1965) Historic littoral cones in Hawaii. Pac Sci ΧΙΧ(3–11)

[CR34] Morrissey MM, Thordarson T (1991). Origin and occurrence of pseudocrater fields in southern Iceland. Eos.

[CR35] Morrisey M, Zimanowski B, Wohletz L, Büttner R (2000) Phreatomagmatic fragmentation. In: Sigurdsson H (ed) Encyclopedia of volcanoes. Academic, London, pp 431–445

[CR36] Pyle DM (1989). The thickness, volume and grainsize of tephra fall deposits. Bull Volcanol.

[CR37] Reynolds P, Brown RJ, Thordarson T, Llewellin EW, Fielding K (2015) Rootless cone eruption processes informed by dissected tephra deposits and conduits. Bull Volcanol 77(72). doi:10.1007/s00445-015-0958-3

[CR38] Rossi MJ (1997). Morphology of the 1984 open-channel lava flow at Krafla Volcano, Northern Iceland. Geomorphol.

[CR39] Rossi MJ, Gudmundsson A (1996). The morphology and formation of flow-lobe tumuli on Icelandic shield volcanoes. J Volcanol Geotherm Res.

[CR40] Schipper CI, Sonder I, Schmid A, White JDL, Dürig T, Zimanowski B, Büttner R (2013). Vapour dynamics during magma—water interaction experiments: hydromagmatic origins of submarine volcaniclastic particles (limu o Pele). Geophys J International.

[CR41] Schmincke H-U (2004). Volcanism.

[CR42] Sheridan MF, Wohletz KH (1981). Hydrovolcanic explosions: the systematics of water–pyroclast equilibration. Science.

[CR43] Sheridan MF, Wohletz KH (1983) Hydrovolcanism: basic considerations and review. In: MF Sheridan and F Barberi (eds.), Explosive volcanism. J Volcanol Geotherm Res 17: 1–29

[CR44] Sinton J, Grönvold K, Sæmundsson K (2005). Postglacial eruptive history of the Western Volcanic Zone, Iceland. Geochem Geophys Geosyst.

[CR45] Terry RD, Chilingar GV (1955). Summary of “Concerning some additional aids in studying sedimentary formations” by M.S. Shuetsov. J Sediment Petrol.

[CR46] Thorarinsson S (1951). Laxargljufur and Laxarhraun: a tephrachronological study. Geogr Ann.

[CR47] Thorarinsson S (1953). The crater groups in Iceland. Bull Volcanol.

[CR48] Thordarson T, Miller DJ, Larsen G (1998). New data on the Leidolfsfell cone group on South Iceland. Jokull.

[CR49] Tómasson J, Thorsteinsson T, Kristmannsdóttir H, Friðleifsson IB (1977) Höfuðborgarscæðið: Jarðhitarannsóknir 1965–73 (Geothermal investigations in Greater Reykjavík), OS-JHD-7703 Report, Reykjavík, Iceland; 109 pp

[CR50] von Komorowicz M (1912) Vulkanologische Studien auf Einigen Inseln des Atlantischen Oceans. E. Schweizerbart'sche Verlagsbuchhandlung. Nägele und Dr. Sproesser, Stuttgart, 189 p. (plus plates and maps)

[CR51] Walker GPL (1991). Structure and origin by injection of lava under surface crust of tumuli, “lava rises”, “lava-rise pits”, and “lava inflation clefts” in Hawaii. Bull Volcanol.

[CR52] White JDL (1996). Impure coolants and interaction dynamics of phreatomagmatic eruptions. J Volcanol Geotherm Res.

[CR53] White JDL, Houghton BF (2006). Primary volcaniclastic rocks. Geology.

[CR54] Wohletz K (1983). Mechanisms of hydrovolcanic pyroclast formation: size, scanning electron microscopy, and experimental studies. J Volcanol Geotherm Res.

[CR55] Wohletz K (1986). Explosive magma-water interactions: thermodynamics, explosion mechanisms, and field studies. Bull Volcanol.

[CR56] Wohletz K (2002). Water/magma interaction: some theory and experiments on peperite formation. J Volcanol Geotherm Res.

[CR57] Wohletz KH, Sheridan MF (1981). Rampart crater ejecta: experiments and analysis of melt-water interactions. Reports of the Planetary Geology Program 1980–1981. NASA Tech Memo.

[CR58] Wohletz KH, Sheridan MF (1983). Hydrovolcanic explosions II. Evolution of basaltic tuff rings and tuff cones. Am J Sci.

[CR59] Zimanowski B (1998) Phreatomagmatic explosions. In: Freundt A, Rosi M (eds) From magma to tephra: modelling physical processes of explosive volcanic eruptions. Elsevier, Amsterdam, pp 25–554

[CR60] Zimanowski B, Fröhlich G, Lorenz V (1991). Quantitative experiments on phreatomagmatic explosions. J Volcanol Geotherm Res.

