# Supplementary material for: Rootless tephra stratigraphy and emplacement processes
Source: Bull Volcanol. 2017 Jan 10;79(1):11. doi: 10.1007/s00445-016-1086-4 (PMC7115078; doi:10.1007/s00445-016-1086-4)
Supplement: Supplementary file 2 — (PDF 472 kb) [file 445_2016_1086_MOESM2_ESM.pdf]

Appendix 1: Rootless tephra stratigraphy and emplacement processes

Christopher W. Hamilton[1] • Erin P. Fitch[2] • Sarah A. Fagents[3] • Thorvaldur Thordarson[4]

[1] Lunar and Planetary Laboratory, University of Arizona, 1629 E. University Blvd. Tucson, AZ 85721, USA
[2] Department of Geology and Geophysics, University of Hawaii, Honolulu, HI, USA
[3] Hawaii Institute of Geophysics and Planetology, University of Hawaii, Honolulu, HI, USA
[4] Faculty and Institute of Earth Sciences, University of Iceland; Reykjavík, Iceland

Deriving mud and basalt mass percentages

Rootless tephra deposits within Rauðhólar include basaltic material and lacustrine sediment (hereafter referred to as "mud"). Therefore, it is necessary to distinguish between these two types of material in our grain-size distributions. Unconsolidated lacustrine sediment mainly occurs within the fine lapilli to very fine ash portion of the deposits, but also occurs as larger indurated "mud clots". The abundance of basaltic clasts and mud in each sample was visually estimated for each 0.5ϕ bin in the range from –3.0 to 4.5ϕ. To convert our estimates of mud volume percentage to mass percentage, the difference in its density relative to basalt was accounted for using the following equation,

$$m_b = M\left(\frac{1}{\left(\frac{v\%_m}{v\%_b}\right)\left(\frac{\rho_m}{\rho_b}\right)+1}\right), \quad (1)$$

where m_b is the mass the basalt fraction within a particular bin, M is the total mass within the bin, $v\%_m$ and $v\%_b$ are the volume percent of mud and basalt, respectively, and ρ_m and ρ_b are the density of mud and basalt, respectively. By measuring the volume percentages of mud and basalt, only the density ratio of mud-to-basalt (ρ_m/ρ_b) is needed to complete this calculation. This ratio was obtained by randomly selecting three samples (R-12-11, R-12-18, and R-12-45) and measuring the physical properties of the two types of componentry within them. The resulting ratios of ρ_m/ρ_b was 0.51 for R-12-11, 0.59 for R-12-18, and 0.56 for R-12-14, which yields an average ratio of 0.55. Given the narrow variability of the ρ_m/ρ_b ratio in these examples, we assumed that this correction factor would be appropriate to use in Eq. 1 when calculating the relative masses of basalt and mud in the other samples based on observed mud abundance.

Grain size corrections

Imperfectly sized mesh was used to make the field sieves employed in 2007 and 2008. The mesh partially impeded the passage of material from the 8 mm (–3ϕ) sieve to the 4 mm (–2ϕ) sieve. To estimate the proportion of material that was affected during the earlier sampling efforts, a second set of samples were collected in 2012 from previously sampled layers. Comparisons of the data revealed three archetypal grain-size histograms, which were used to obtain correction factors for older samples. To obtain the correction factors, the difference between the –3ϕ mass fraction in the 2012 sample (i.e., –3ϕ mass / total mass) was subtracted from the mass fraction in the –3ϕ bin for corresponding sample from 2007 or 2008. The difference was then used to estimate the mass of material that should have passed through the –3ϕ sieve to the –2 ϕ bin. For the older samples, this mass was subtracted from the –3ϕ bin and added to the –2ϕ bin. The correction factors that we calculated for the –3ϕ bin of the Type 1, 2, and 3 samples were 0.48, 0.55, and 0.37, respectively. Affected samples from 2007 and 2008 include: Type 1 (R-07-03, R-07-05, R-07-07, R-07-09, R-07-12, R-07-24B, R-07-25, and R-08-18); Type 2 (R-07-01, R-07-02, R-07-04, R-07-06, R-07-08, R-07-10, R-07-11, R-07-18, R-07-19, R-07-21, R-07-22, and R-08-17); and Type 3 (R-07-16). The accompanying spreadsheet provides all grain-size data used in this study.
